# Mutations in *NAKED-ENDOSPERM* IDD genes reveal functional interactions with *SCARECROW* during leaf patterning in C_4_ grasses

**DOI:** 10.1371/journal.pgen.1010715

**Published:** 2023-04-17

**Authors:** Thomas E. Hughes, Olga Sedelnikova, Mimi Thomas, Jane A. Langdale

**Affiliations:** Department of Biology, University of Oxford, Oxford, England; "USDA-ARS Pacific West Area", UNITED STATES

## Abstract

Leaves comprise a number of different cell-types that are patterned in the context of either the epidermal or inner cell layers. In grass leaves, two distinct anatomies develop in the inner leaf tissues depending on whether the leaf carries out C_3_ or C_4_ photosynthesis. In both cases a series of parallel veins develops that extends from the leaf base to the tip but in ancestral C_3_ species veins are separated by a greater number of intervening mesophyll cells than in derived C_4_ species. We have previously demonstrated that the GRAS transcription factor SCARECROW (SCR) regulates the number of photosynthetic mesophyll cells that form between veins in the leaves of the C_4_ species maize, whereas it regulates the formation of stomata in the epidermal leaf layer in the C_3_ species rice. Here we show that SCR is required for inner leaf patterning in the C_4_ species *Setaria viridis* but in this species the presumed ancestral stomatal patterning role is also retained. Through a comparative mutant analysis between maize, setaria and rice we further demonstrate that loss of NAKED-ENDOSPERM (NKD) INDETERMINATE DOMAIN (IDD) protein function exacerbates loss of function *scr* phenotypes in the inner leaf tissues of maize and setaria but not rice. Specifically, in both setaria and maize, *scr;nkd* mutants exhibit an increased proportion of fused veins with no intervening mesophyll cells. Thus, combined action of SCR and NKD may control how many mesophyll cells are specified between veins in the leaves of C_4_ but not C_3_ grasses. Together our results provide insight into the evolution of cell patterning in grass leaves and demonstrate a novel patterning role for IDD genes in C_4_ leaves.

## Author summary

The correct patterning of cell-types within organs is required to ensure appropriate morphology and physiology in any organism. In grasses, two distinct cellular patterns develop in internal leaf tissues depending on the type of photosynthesis that is carried out. Previous work suggested that the transcription factor SCARECROW (SCR) has different patterning roles in leaves of rice and maize which carry out ‘C_3_’ versus ‘C_4_’ photosynthesis, respectively. *SCR* patterns epidermal cell-types in rice leaves and internal leaf tissues in maize. Here we generate *scr* mutations in another C_4_ plant, *Setaria viridis*, and reveal patterning defects in both the epidermis and inner leaf tissues. This observation provides information about the evolutionary trajectory of the SCR patterning pathway in grasses but also raises the question of how the inner leaf patterning component is distinguished from the epidermal role. Through the identification of genetic interactions between SCR and a second transcription factor NAKED-ENDOSPERM (NKD) we demonstrate that combined SCR/NKD function patterns inner leaf tissues in the C_4_ grasses maize and *S*. *viridis* but not in the C_3_ grass rice. We propose that patterning of epidermal cell-types represents the ancestral role for SCR in grass leaves and that as C_4_ grasses evolved, the gene was recruited along with NKD to pattern inner leaf cell types. Some C_4_ species have retained the ancestral function whereas others such as maize have not.

## Introduction

Understanding how cell patterning is genetically regulated is a key challenge in developmental biology. In grass leaves, two distinct cellular anatomies underpin photosynthesis. In grasses such as rice (*Oryza sativa*) that carry out C_3_ photosynthesis, widely spaced parallel veins are encircled by non-photosynthetic bundle-sheath (BS) cells which are themselves separated by up to ten photosynthetic mesophyll (M) cells. By contrast, in grasses that perform C_4_ photosynthesis, such as maize (*Zea mays*) and green foxtail (*Setaria viridis*), parallel veins are surrounded by concentric layers of BS and M cells, both of which are photosynthetic. This arrangement of cell-types is referred to as ‘Kranz’ because the two cell-types form wreaths around the veins and Kranz is German for wreath [1]. Notably Kranz anatomy evolved from C_3_-type anatomy on multiple independent occasions [2,3], each occurrence generating leaves with higher vein densities than in the ancestral form because BS cells are separated by fewer M cells. To date, very few regulators of cell-patterning in inner leaf tissues have been identified in C_3_ or C_4_ grass species.

We previously demonstrated that duplicate homeolog genes encoding the GRAS transcription factor SCARECROW (SCR) regulate cell divisions in the innermost ground meristem layer of maize leaf primordia to determine the number of M cells that form between veins [4]. In double *Zmscr1;Zmscr1h* (where *h* indicates the homeologous gene copy) mutants, the majority of BS cells are separated by one rather than two M cells, and in some cases BS cells are fused with no intervening M cells. In addition, many veins develop ectopic sclerenchyma either ad- or abaxially and some veins are surrounded by additional BS cells that are not in contact with the vasculature. Intriguingly, no such patterning perturbations were observed in inner leaf tissues when *SCR* orthologs were mutated in rice [5]. Instead, loss of function mutants in rice fail to develop stomata on the leaf surface [5,6]. The distinction between mutant phenotypes in maize and rice leaves raises the possibility that the deployment of SCR function in inner leaf tissues was associated with the evolution of Kranz anatomy, with the patterning of stomata in the epidermis being the ancestral role in grass leaves.

Although the function of SCR itself could differ between maize and rice, for example by targeting different downstream genes, the distinct patterning roles observed could alternatively result from species-specific differences in SCR interacting proteins. Little is known about how SCR-mediated patterning is regulated in monocots but in Arabidopsis roots, SCR functions with another GRAS transcription factor (SHORTROOT (SHR)) and with several INDETERMINATE DOMAIN (IDD) C2H2 zinc-finger transcription factors [7–11]. The IDD genes (also referred to as BIRD genes), act both to modulate *SCR* and *SHR* gene expression and to co-regulate the expression of downstream target genes [9]. Despite the existence of many IDD genes in grass genomes, and the demonstration that *SCR* and *SHR* have patterning functions in maize and rice, links between *SCR* and IDD genes in the patterning of either root or leaf cell-types in monocots have not yet been established.

We previously proposed that the *NAKED-ENDOSPERM* (*NKD*) *IDD* genes (originally referred to as *ZmJAY* genes) may function during Kranz patterning in maize [12,13]. This proposal was based on the fact that *ZmNKD1* and *ZmNKD2* transcripts accumulate during early leaf development at the relevant stages of Kranz patterning [14], both transcripts accumulate specifically in mesophylls of mature leaves [15,16] and both have known patterning functions in aleurone development [17,18]. Building on this suggestion and on the outstanding questions raised above, here we have tested two hypotheses. First, that SCR function is required for patterning inner leaf tissues in C_4_ grasses and second that NKD is a component of the patterning pathway. By characterizing *scr* mutants in the C_4_ species *Setaria viridis* and carrying out a comparative analysis of *scr;nkd* mutants in maize, *Setaria viridis* and rice, we found evidence to support both hypotheses.

## Results

### SCR patterns both epidermal and inner tissues in leaves of *Setaria viridis*

**Fig 1:**
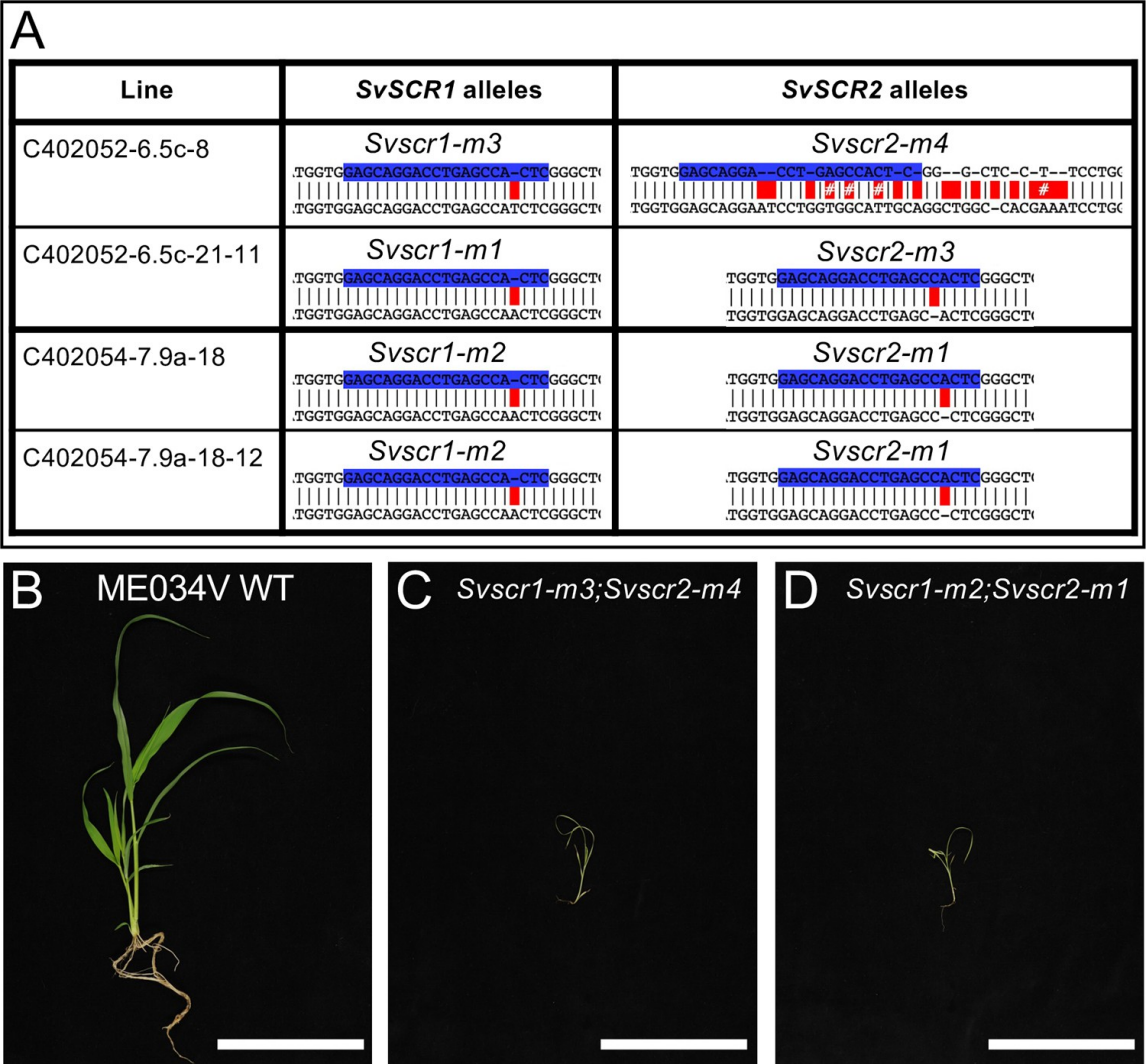
Mutations in *SvSCR1* and *SvSCR2* affect plant growth. **A)** Sequences of mutant *Svscr1* and *Svscr2* alleles in two independent lines (C402052-6 & C402054-7). Wild-type (WT) sequence is shown on top, with the sequence of the mutant allele shown beneath. Guide sequences are depicted in blue, and mismatches between the WT and mutant sequence indicated in red. **B-D)** Photos of WT ME034V (B), *Svscr1-m3;Svscr2-m4* (C) and *Svscr1-m2-Svscr2-m1* (D) plants taken 20 days after sowing. Scale bars: 10cm.

**Fig 2:**
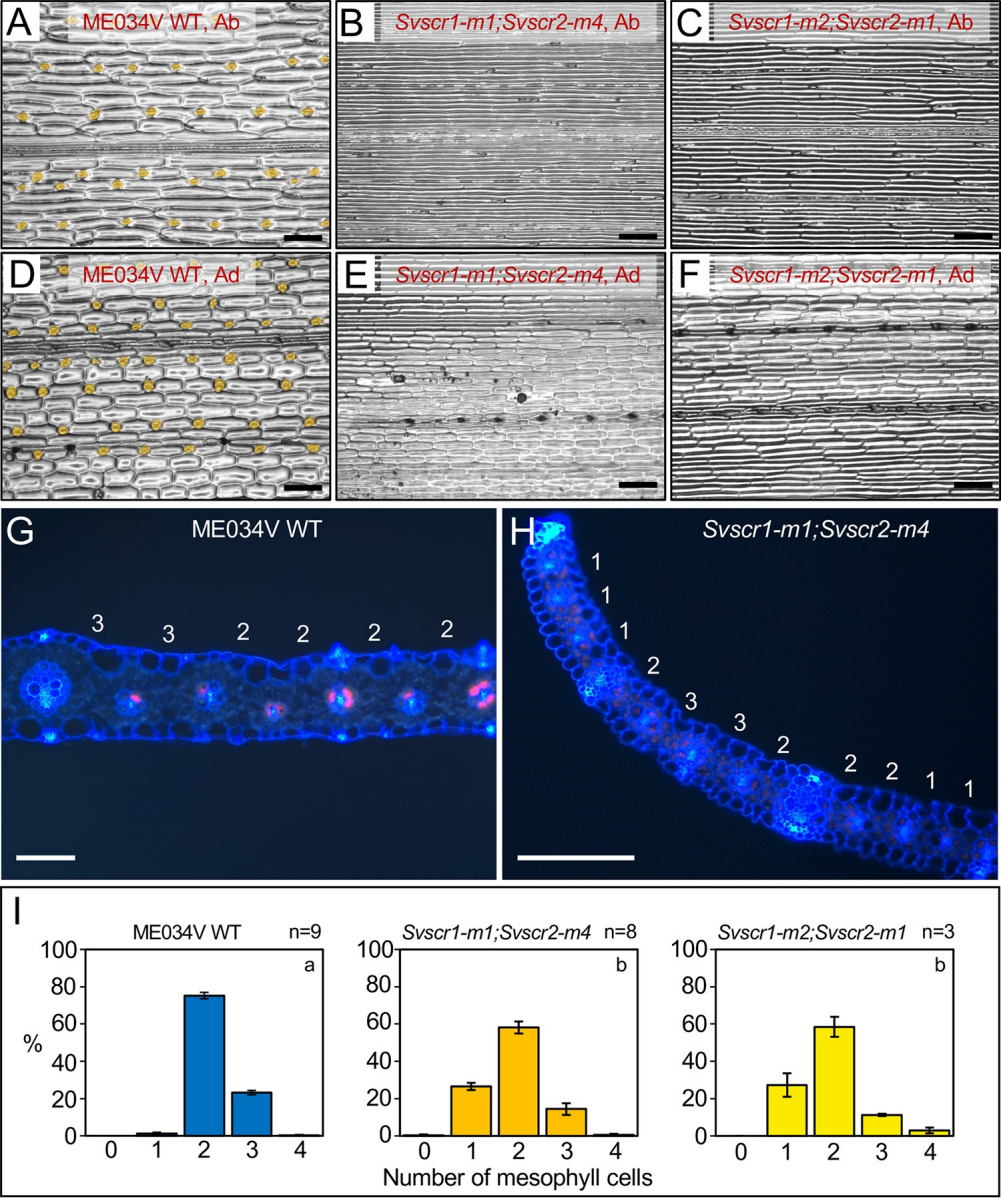
*Svscr1;Svscr2* mutant plants have no stomata and fewer mesophyll cells separating veins. **A-F)** Stomatal impressions of wild-type (WT) ME034V (A,D), *Svscr1-m1;Svscr2-m4* (B,E) and *Svscr1-m2;Svscr2-m1* (C,F) leaf 3 from either the abaxial (A-C) or adaxial (D-F) surface. Stomata are false coloured orange. Scale bars: 100μm. **G-H)** Transverse sections imaged using UV illumination of WT ME034V (G) and *Svscr1-m1;Svscr2-m4* (H) leaf 4, taken from the mid-point along the proximal-distal axis. The number of mesophyll cells between each pair of veins is indicated above the leaf. Scale bars: 100μm. **I)** Histograms summarizing the mean number of mesophyll cells separating veins in WT ME034V and two independent *Svscr1;Svscr2* mutants. The number of biological replicates is indicated above each plot and letters in the top right corner of each plot indicate statistically different groups (*P*≤0.05, one-way ANOVA and Tukey’s HSD) calculated using the mean number of mesophyll cells in each genotype (raw data in S1 Table).

To determine whether the distinct patterning roles of SCR in the rice epidermis versus the maize inner leaf reflect C_3_- versus C_4_-specific functions, we generated loss-of-function mutants in the C_4_ species *Setaria viridis* (hereafter referred to as setaria) using CRISPR (Figs 1A and S1). As in maize and rice, two *SCR* genes are present in setaria (*SvSCR1*- Sevir.7G316501 and *SvSCR2*- Sevir.8G008100), each pair the result of a within species duplication [4]. In gene edited T2 lines, a proportion of plants were identified that were extremely stunted, paler than wild-type and did not survive beyond 3–4 weeks after germination (Fig 1B–1D). These plants appeared at the expected segregation ratios of 1/4 or 1/16 depending on whether the parental plant was *Svscr1/+*;*Svscr2* (or *Svscr1*;*Svscr2/+*) or *Svscr1/+*;*Svscr2/+* respectively. In all cases, these phenotypically abnormal plants were confirmed to be homozygous for both *Svscr1* and *Svscr2*, with phenotypically wild-type plants always being heterozygous or wild-type for one of the *SCR* genes. Because single *Svscr* mutants displayed no growth perturbations (S2 Fig) and single mutants in both rice and maize were not associated with any patterning defects, all further analyses were undertaken with homozygous double mutants. The perturbed growth phenotype exhibited by *Svscr1;Svscr2* plants was far more severe than that seen in maize *Zmscr1;Zmscr1h* mutants (where plants can be grown for 6–8 weeks in the greenhouse without issue) [4], but was similar to the phenotype observed in *Osscr1*;*Osscr2* mutants of rice [5]. We therefore reasoned that, as in rice, *SCR* may play a role in stomatal patterning in setaria. Epidermal impressions of *Svscr1;Svscr2* mutant leaves confirmed this hypothesis, revealing an absence of stomata on both the abaxial and adaxial leaf surfaces (Fig 2A–2F). Therefore, the stomatal patterning role first identified in rice is not a C_3_-specific function.

To determine whether *SCR* also plays a role in inner leaf patterning in setaria, transverse sections of wild-type and *Svscr1;Svscr2* mutant leaves were examined (Fig 2G and 2H). In ME034V wild-type leaves, around 70–80% of veins were separated by two M cells with the rest separated by three (a feature that is more common in setaria than in maize) (Fig 2I). In contrast, 20–30% of veins in *Svscr1;Svscr2* mutant leaves were separated by just a single M cell (Fig 2I). These data demonstrate that, as in maize, *SvSCR* genes regulate cell divisions in the ground meristem to determine how many M cells develop between BS cells. Intriguingly, *SvSCR* genes undertake this inner leaf patterning role in addition to a role in stomatal patterning.

### A feedback loop may link *SCR* and *NKD* IDD gene expression in maize

**Fig 3:**
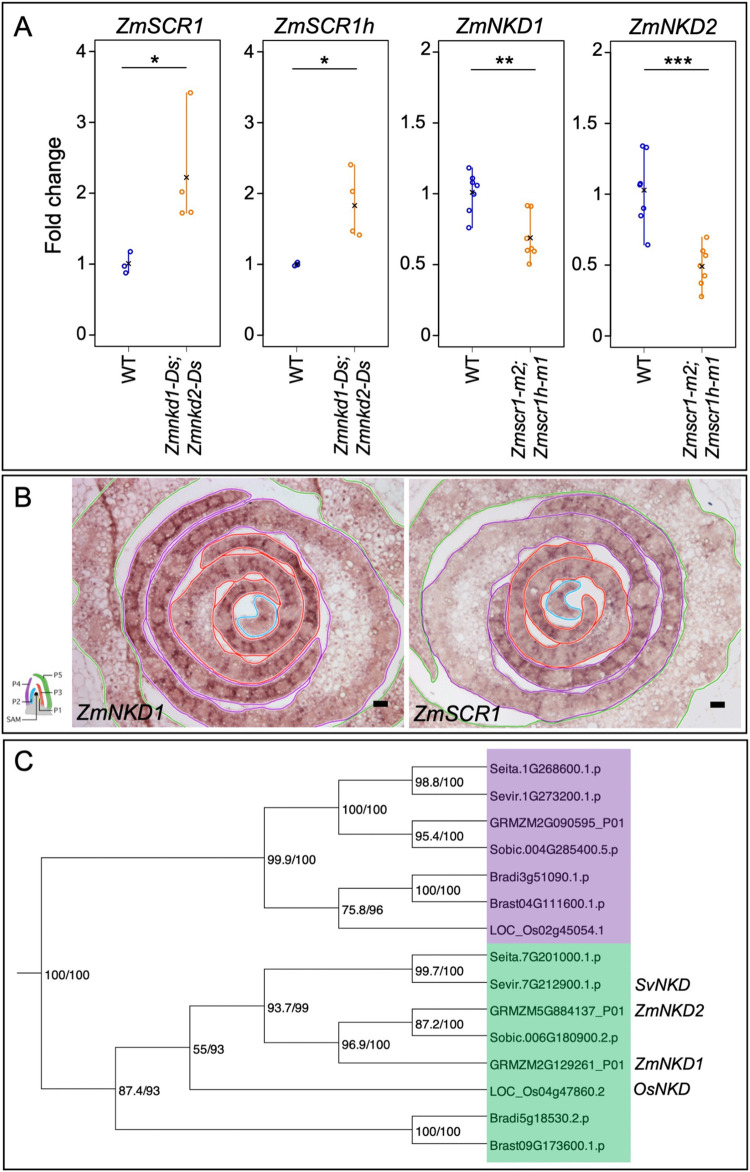
*NKD* and *SCR* transcripts accumulate in the same spatial domain with levels determined by a feedback loop. **A)** Quantitative RT-PCR of *ZmSCR1* and *ZmSCR2* transcripts in the *Zmnkd1-Ds;Zmnkd2-Ds* mutant, and *ZmNKD1* and *ZmNKD2* in the *Zmscr1-m2;Zmscr1h-m2* mutant. Open circles are individual biological replicates, black crosses indicate the mean for each genotype. Statistical significance as calculated on log-transformed fold change data by Welch’s *t*-test (two-tailed) indicated above each plot: *P≤0.05; **P≤0.01; ***P≤0.001 (raw data in S1 Table). **B)** *In-situ* hybridization to *ZmNKD1* and *ZmSCR1* in maize wild-type B73 apices. In each image the P2 primordium is outlined in blue, P3 in red, P4 in purple and P5 in green, as indicated in the adjacent cartoon diagram. Darker purple signal represents successful hybridization to each transcript of interest. Scale bars: 50μm. **C)** Maximum likelihood phylogeny of the *NKD* genes in monocots. The *NKD* clade is highlighted in green, and the adjacent monocot clade in purple. Bootstrap values are displayed at each branch of the phylogeny.

The distinct roles of *SCR* genes in maize, rice and setaria–patterning inner, epidermal or both tissue layers of the leaf respectively–could be due to species-specific differences in the activity of interacting IDD genes, of which *NKD* genes are potential candidates. To determine whether the expression of *ZmSCR1/h* genes is interconnected with *ZmNKD1/2* gene function in maize (as is the case for *SCR* and IDD genes in Arabidopsis), we first quantified transcript levels of each gene in existing double *Zmscr1-m2;Zmscr1h-m1* [4] and double *Zmnkd1-Ds;Zmnkd2-Ds* [17] mutants (Figs 3A and S3). *ZmSCR1* and *ZmSCR1h* transcripts accumulated at elevated levels in the *Zmnkd1-Ds;Zmnkd2-Ds* mutant, in both cases being increased around two-fold relative to wild-type (Fig 3C). In contrast, *ZmNKD1* and *ZmNKD2* transcripts accumulated at lower levels in *Zmscr1-m2;Zmscr1h-m1* mutants than in wild-type, with *ZmNKD1* reduced on average to 60% and *ZmNKD2* to 50% of wild-type levels (Fig 3A). These differences are quite modest but the role of both proteins as transcription factors could allow small fold differences to be amplified downstream. Such a scenario is particularly plausible if gene expression is restricted to just a subset of cells in the tissue. To determine whether this is the case, *in situ* hybridization was carried out to localize transcripts in developing leaf primordia. Fig 3B shows that both *NKD1* and *SCR1* transcripts preferentially accumulate in the ground meristem cells that surround developing vascular centres. Together these data suggest that a negative feedback loop (either direct or indirect) may influence *ZmSCR1/1h* and *ZmNKD1/2* transcript levels, with ZmSCR1/ZmSCR1h positively affecting the expression of *ZmNKD1*/*ZmNKD2*, and ZmNKD1/ZmNKD2 negatively affecting the expression of *ZmSCR1*/*ZmSCR1h*.

### Loss of function *nkd* mutants do not exhibit perturbed leaf development

**Fig 4:**
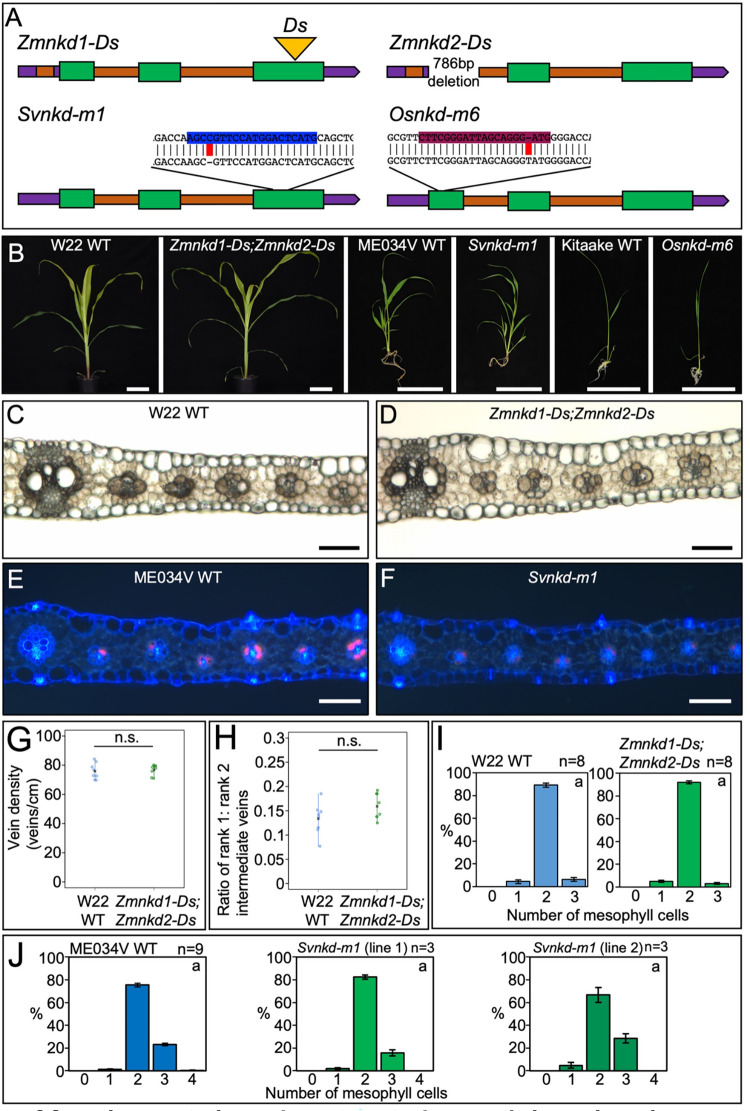
*nkd* loss of function mutations do not perturb growth in maize, rice or setaria. **A)** Cartoon depictions of loss of function *nkd* alleles in maize (*Zm*), setaria (*Sv*) and rice (*Os*). In each case, 5’ and 3’ untranslated regions are depicted in purple, introns in orange and coding regions in green. Transposon insertions are indicated by a yellow triangle. For setaria and rice, CRISPR guide sequences are indicated in blue and maroon respectively, with the sequence of the mutant allele beneath with edits highlighted in red. **B)** Whole plant phenotypes of maize, setaria and rice *nkd* mutants. Photos were taken 31 days (maize), 20 days (setaria) or 14 days (rice) after sowing. Scale bars: 10cm. **C-F)** Transverse sections of maize wild-type (WT) W22 (C), *Zmnkd1-Ds;Zmnkd2-Ds* (D), setaria WT ME034V (E) and *Svnkd-m1* (F), imaged under either brightfield (maize) or UV illumination (setaria). Images were taken at the mid-point along the proximal-distal axis of either leaf 5 (maize) or leaf 4 (setaria). Scale bars: 100μm. **G-H)** Quantification of vein density (G) and the ratio of rank1:rank2 intermediate veins (H) of WT W22 (blue) and *Zmnkd1-Ds;Zmnkd-Ds* (green) mutants. Open circles indicate distinct biological replicates, black crosses indicate the mean for each genotype. Statistical significance as calculated by Welch’s *t*-test (two-tailed) indicated above each plot: n.s. *P*>0.05 (raw data in S1 Table). **I-J)** Histograms summarising the mean number of mesophyll cells separating veins in WT W22 versus *Zmnkd1-Ds;Zmnkd2-Ds* (I) and WT ME034V versus two independent *Svnkd-m1* lines (J). Error bars are the standard error of the mean, and the number of biological replicates is indicated above each plot. Letters in the top right corner of each plot indicate statistically different groups (*P*≤0.05, one-way ANOVA and Tukey’s HSD) calculated using the mean number of mesophyll cells in each genotype (raw data in S1 Table). WT is coloured blue and *nkd* mutants green.

Given the patterning role of SCR in leaves of maize, setaria and rice, and the interaction between *SCR* and *NKD* gene expression in maize, we next sought to determine the role of *NKD* in leaf development in each of the three species. To this end, we first identified *NKD* orthologs in setaria and rice by constructing a maximum likelihood phylogeny. Fig 3C shows that *NKD* exists as a single copy gene in both setaria and rice (*SvNKD—*Sevir.7G212900, *OsNKD—*LOC_Os04g47860), suggesting that the two copies in maize (*ZmNKD1* and *ZmNKD2*) resulted from the recent whole genome duplication [19]. It is possible that the two copies of *NKD* in maize have undergone functional divergence, however, given that they function redundantly in the context of seed development [17], and that the majority of maize homeolog gene pairs have matching expression profiles in developing leaves [20], it is most parsimonious that they function redundantly during leaf development. For phenotypic characterization, the homozygous double *Zmnkd1-Ds;Zmnkd2-Ds* line and newly generated CRISPR loss-of-function mutants in setaria and rice were all compared to wild-type lines from the same genetic background (Figs 4A, S1 and S4). Homozygous *Zmnkd1-Ds;Zmnkd2-Ds* seed exhibited the characteristic shrunken kernel phenotype that is caused by defective patterning of the aleurone layer but no altered seed phenotype was observed in *Svnkd* or *Osnkd* mutants (S5 Fig). Overall plant growth was normal in maize, setaria and rice *nkd* mutants (Fig 4B) and no leaf patterning perturbations were observed (Figs 4C–4J and S6). In *Zmnkd1-Ds;Zmnkd2-Ds* mutants, no changes relative to wild-type were observed in leaf vein-density or in the ratio of rank-1 to rank-2 intermediate veins (Fig 4G and 4H), traits that are both altered in *Zmscr1;Zmscr1h* mutants. Furthermore, there was no change in the number of M cells separating veins (the most penetrant phenotype in equivalent *scr* mutants) in either maize or setaria *nkd* mutants (Fig 4I and 4J). Taken together, these results indicate that *NKD* is not necessary for normal leaf development in maize, setaria or rice.

### In maize and setaria, but not in rice, *nkd* loss of function mutations enhance *scr* mutant leaf phenotypes

**Fig 5:**
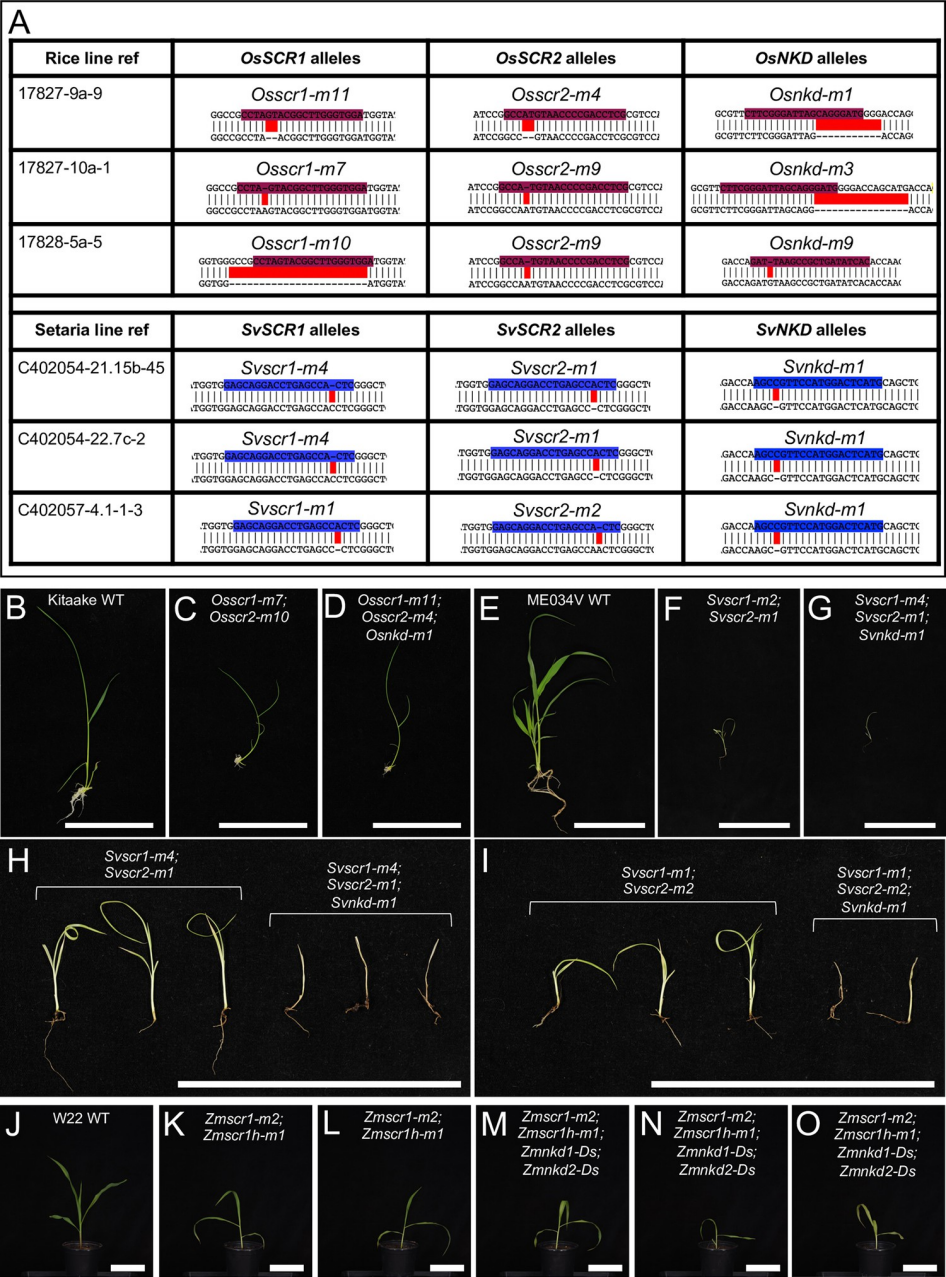
Loss of function *nkd* mutations enhance growth perturbations in *scr* mutants of setaria but not rice. **A)** Sequences of mutant *scr1;scr2;nkd* alleles in three independent lines of both rice and setaria. Wild-type sequences are shown above with guides highlighted in either maroon (rice) or blue (setaria). Edits are highlighted in red. Note *Svscr1-m2* allele is included in Fig 1. **B-O)** Whole plant phenotypes of rice (B-D), setaria (E-I) and maize (J-O). Images were taken 14, 20 or 21 days after sowing for rice, setaria and maize respectively. Plants in (H) and (I) are from segregating families in each case, such that *Svscr1;Svscr2* and *Svscr1;Svscr2;Svnkd* in each panel are siblings. Scale bars: 10cm.

To determine whether a role for *NKD* in leaf patterning could be revealed in the absence of SCR function, quadruple *scr1;scr1h;nkd1;nkd2* mutants of maize and triple *scr1;scr2;nkd* mutants of setaria and rice were generated (Figs 5, S1 and S4). An initial assessment of overall growth phenotypes revealed that perturbations in three independent triple *Osscr1;Osscr2;Osnkd* mutant lines were similar to those seen in double *Osscr1;Osscr2* mutants (Fig 5B–5D). By contrast, triple *Svscr1;Svscr2;Svnkd* mutants displayed more severe perturbations than double *Svscr1;Svscr2* mutants (Fig 5E–5I). In the progeny of *Svscr1;Svscr2/+;Svnkd/+* plants from two independent lines, the resultant triple mutants were smaller and died faster than double *Svscr1;Svscr2* mutant siblings (Fig 5H and 5I). This was consistent in lines fixed for *Svnkd*, where only 8/20 and 8/34 triple mutants from two independent *Svscr1;Svscr2;Svnkd* lines survived 22 days after sowing, compared to 14/15 and 13/17 *Svscr1;Svscr2* double mutants. In maize, quadruple *Zmscr1-m2;Zmscr1h-m1;Zmnkd1-Ds;Zmnkd2-Ds* mutants appeared similar to double *Zmscr1-m2;Zmscr1h-m1* mutants, with pale, drooping leaves and a shorter stature than either wild-type or double *Zmnkd1-Ds;Zmnkd2-Ds* mutant plants (Fig 5J–5O).

**Fig 6:**
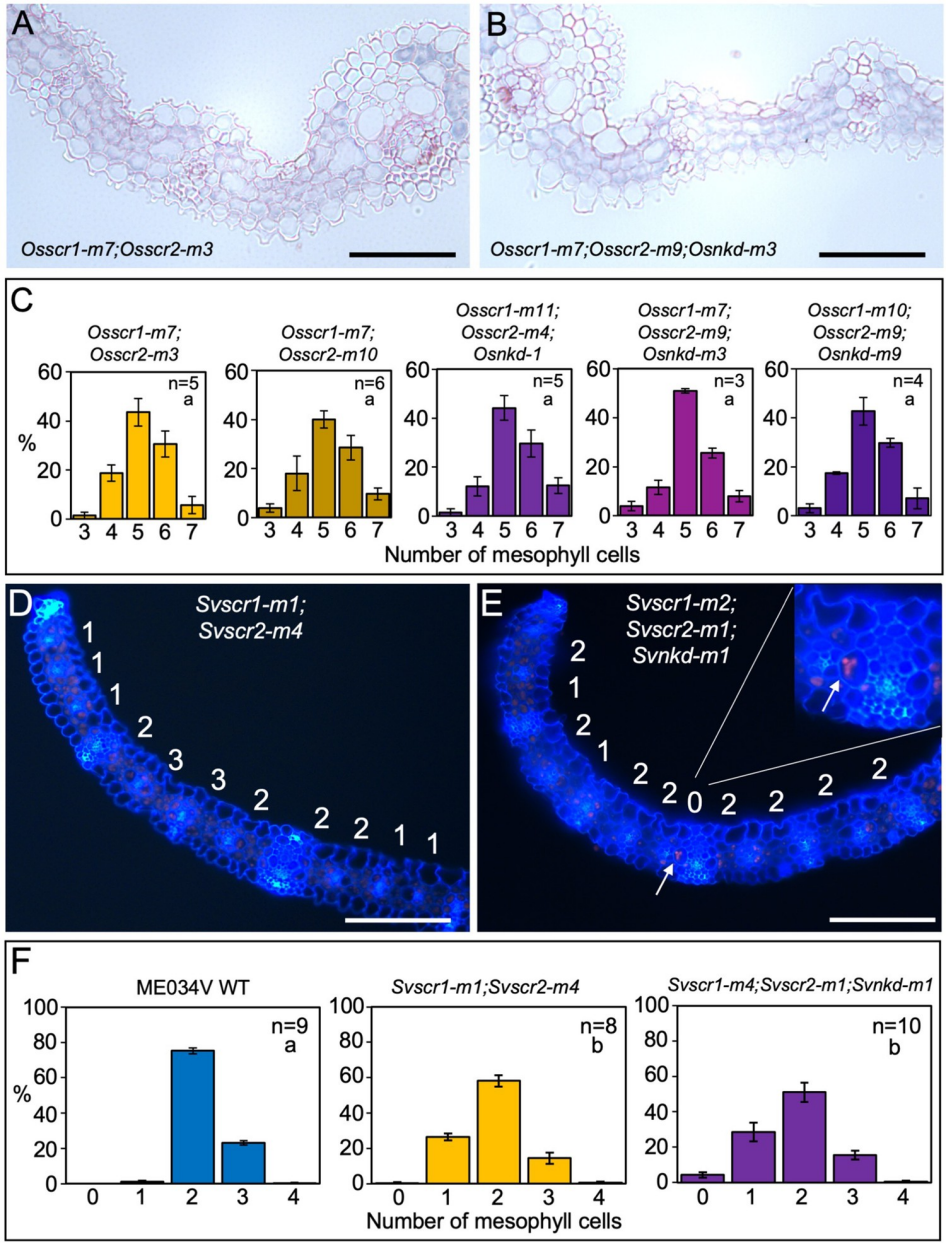
Loss of function *nkd* mutations induce the formation of fused leaf veins in *scr* mutants of setaria but not rice. **A-B)** Transverse sections of *Osscr1-m7;Osscr2-m3* (A) and *Osscr1-m7;Osscr2-m9;Osnkd-m3* (B) mutant leaves, taken at the midpoint along the proximal-distal axis of leaf 5. **C)** Histograms summarizing the mean number of mesophyll cells separating veins in two independent *Osscr1;Osscr2* (yellow) and three independent *Osscr1;Osscr2;Osnkd* (purple) mutant lines. Error bars are standard error of the mean (raw data in S1 Table). Sample sizes (n =) are biological replicates. **D-E)** Transverse sections of *Svscr1-m1;Svscr2-m4* (D) and *Svscr1-m2;Svscr2-m1;Svnkd-m1* (E) mutant leaves, taken at the midpoint along the proximal-distal axis of leaf 4, imaged under UV illumination. The number of mesophyll cells separating veins is displayed above each region. In (E) white arrows and the inset show an example of a fused vein with no separating mesophyll cells. **F)** Histograms summarizing the mean number of mesophyll cells separating veins in wild-type (WT) ME034V (blue), *Svscr1-m1;Svscr2-m4* (yellow) and *Svscr1-m4;Svscr2-m1;Svnkd-m1* (purple) mutants. Error bars are standard error of the mean. Sample sizes (n =) are biological replicates and letters in the top right corner (beneath sample sizes) of each plot indicate statistically different groups (*P*≤0.05, one-way ANOVA and Tukey’s HSD) calculated using the mean number of mesophyll cells in each genotype (raw data in S1 Table). Scale bars: 100 μm.

**Fig 7:**
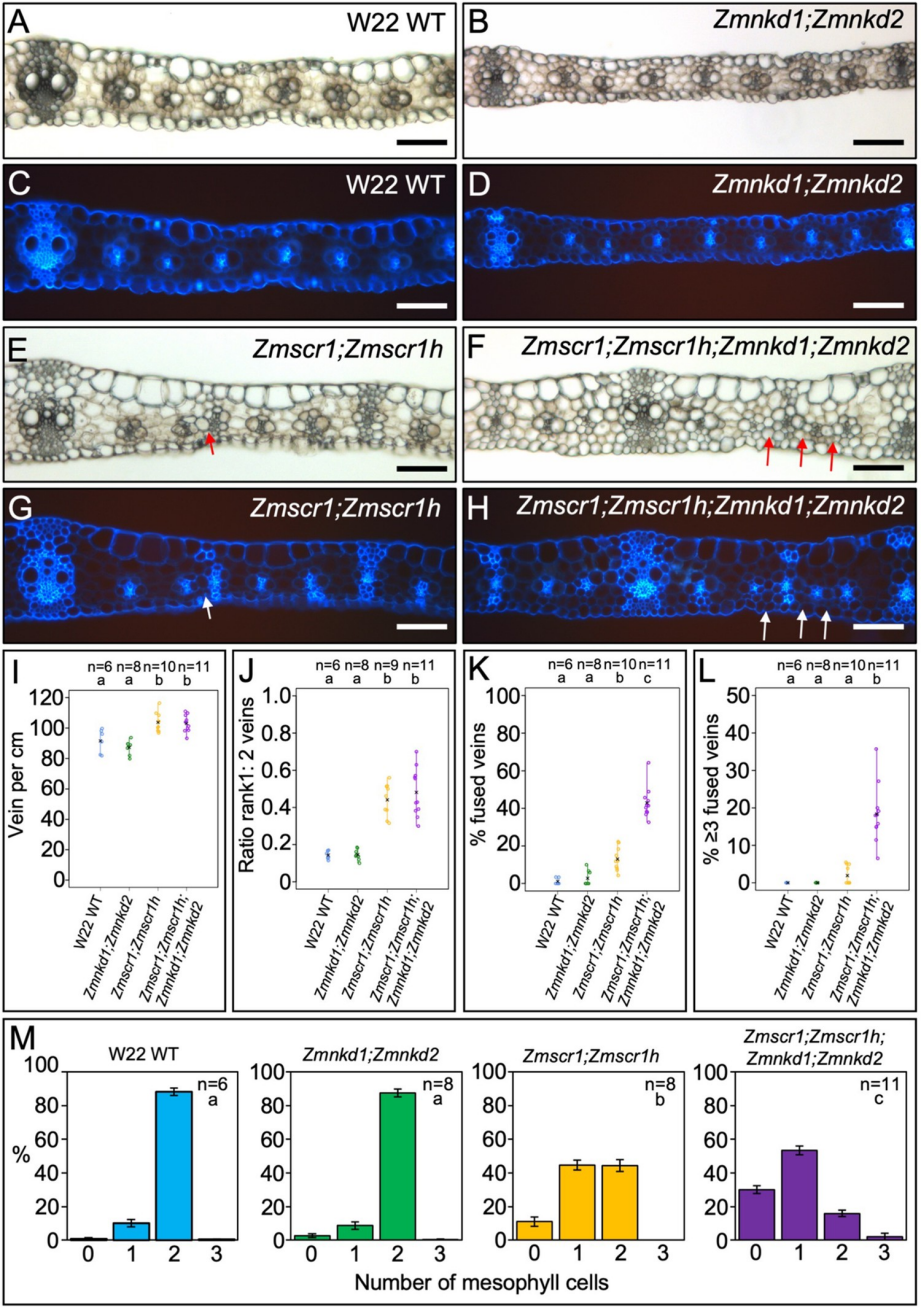
*Zmscr1;Zmscr1h;Zmnkd1;Zmnkd2* quadruple mutants have a striking increase in the number of fused leaf veins compared to *Zmscr1;Zmscr1h* double mutants. **A-H)** Transverse cross sections of wild-type (WT) W22 (A & C), *Zmnkd1-Ds;Zmnkd2-Ds* (B & D), *Zmscr1-m2;Zmscr1h-m1* (E & G) and *Zmscr1-m2;Zmscr1h-m1;Zmnkd1-Ds;Zmnkd2-Ds* (F & H) leaf 3, from the mid-point along the proximal distal axis and imaged under either brightfield (A-B, E-F) or UV (C-D, G-H) illumination. Quadruple mutants were segregating in progeny of selfed *Zmscr1-m2;Zmscr1h-m1/+;Zmnkd1-Ds;Zmnkd2-Ds* parents, *nkd* double mutants were derived from selfed *Zmnkd1-Ds;Zmnkd2-Ds* parents and *scr* double mutants from selfed *Zmscr1-m2/+;Zmscr1h-*m1 parents. Arrows point to fused veins. Scale bars: 100 μm. **I-L)** Strip charts summarizing quantification of vein density (I), the ratio of rank 1: rank 2 intermediate veins (J), the % of fused veins (K) and the % of veins formed in runs of ≥3 fused veins (L). Open circles indicate measurements from independent biological replicates, and black crosses indicate the mean for each genotype. The number of biological replicates (n =) is indicated above each plot and letters at the top of each plot indicate statistically different groups (*P*≤0.05, one-way ANOVA and Tukey’s HSD) (raw data in S1 Table). **M)** Histograms summarizing the mean number of mesophyll cells separating veins in WT W22 (blue), *Zmnkd1-Ds;Zmnkd2-Ds* (green), *Zmscr1-m2;Zmscr1h-m1* (yellow) and *Zmscr1-m2;Zmscr1h-m1;Zmnkd1-Ds;Zmnkd2-Ds* (purple) mutants. Error bars are standard error of the mean. Sample sizes (n =) are biological replicates and letters in the top right corner (beneath sample sizes) of each plot indicate statistically different groups (*P*≤0.05, one-way ANOVA and Tukey’s HSD) calculated using the mean number of mesophyll cells in each genotype (raw data in S1 Table).

To determine the impact of SCR and NKD interactions on leaf development, inner leaf patterning was examined in quadruple (maize) and triple (setaria and rice) mutants. Notably, the complete penetrance of the stomatal phenotype in double *scr1;scr2* mutants of setaria and rice precluded an assessment of interactions with *NKD* during stomatal development but no changes in stomatal patterning were observed in single *Svnkd* or *Osnkd* mutants (S6 Fig). In inner leaf tissues, the number of M cells between veins were the same in three independent triple *Osscr1;Osscr2;Osnkd* mutant lines of rice as in corresponding double *Osscr1;Osscr2* mutants, with the modal number of M cells being five in all cases (Fig 6A–6C). By contrast, fused veins (whereby the BS cells of adjacent veins are in contact with no intervening M cells) were observed in triple *Svscr1;Svscr2;Svnkd* mutants of setaria but not in double *Svscr1;Svscr2* mutants (Fig 6D–6F). Such fusions were not found in every triple mutant examined, with examples observed in 10/22 samples across two experiments with two independent lines (Figs 6, S7 and S8). However, only one occurrence of a fused vein was observed in twenty-five double *Svscr1;Svscr2* mutant samples. Fused veins were also observed in quadruple mutants of maize (Fig 7A–7H). Quadruple mutants did not exhibit higher vein density or an increase in veins associated with sclerenchyma when compared to double *Zmscr1-m2;Zmscr1h-m1* mutants (Fig 7I and 7J) but the increase in the number of veins fused to adjacent veins was consistent and statistically significant (Fig 7F, 7H and 7K–7M). Furthermore, whereas only two adjacent veins fused in double *Zmscr1-m2;Zmscr1h-m1* mutants, groups of three or more fused veins were often seen in quadruple mutants (Fig 7L). These fused veins resulted in a large increase in the number of veins separated by no M cells, with only ~20% of veins separated by the normal two M cells in quadruple mutants, compared to just over 40% in double *Zmscr1-m2;Zmscr1h-m1* mutants. In both maize and setaria the proportion of veins separated by only one M cell was similar in double and quadruple/triple mutants (Figs 6F and 7M). To ensure that these differences were not caused by heterozygosity at unlinked loci following the cross between the *Zmscr1-m2;Zmscr1h-*m1 and *Zmnkd1-Ds;Zmnkd2-D* double mutants, we also compared *Zmscr1-m2;Zmscr1h-m1* mutants pre- and post- outcross and found no difference in the proportion of fused veins (S9 Fig). Furthermore, we validated that the phenotype was consistent across two further quadruple mutants from crosses using independent *Zmnkd1-Ds;Zmnkd2-Ds* plants (S9B Fig). Together these results demonstrate that in the C_4_ species maize and setaria, but not in the C_3_ species rice, *NKD* genes function with *SCR* to determine whether, and how many, M cells are positioned between veins.

### Evidence for a NKD-mediated effect on leaf patterning during embryogenesis in maize

**Fig 8:**
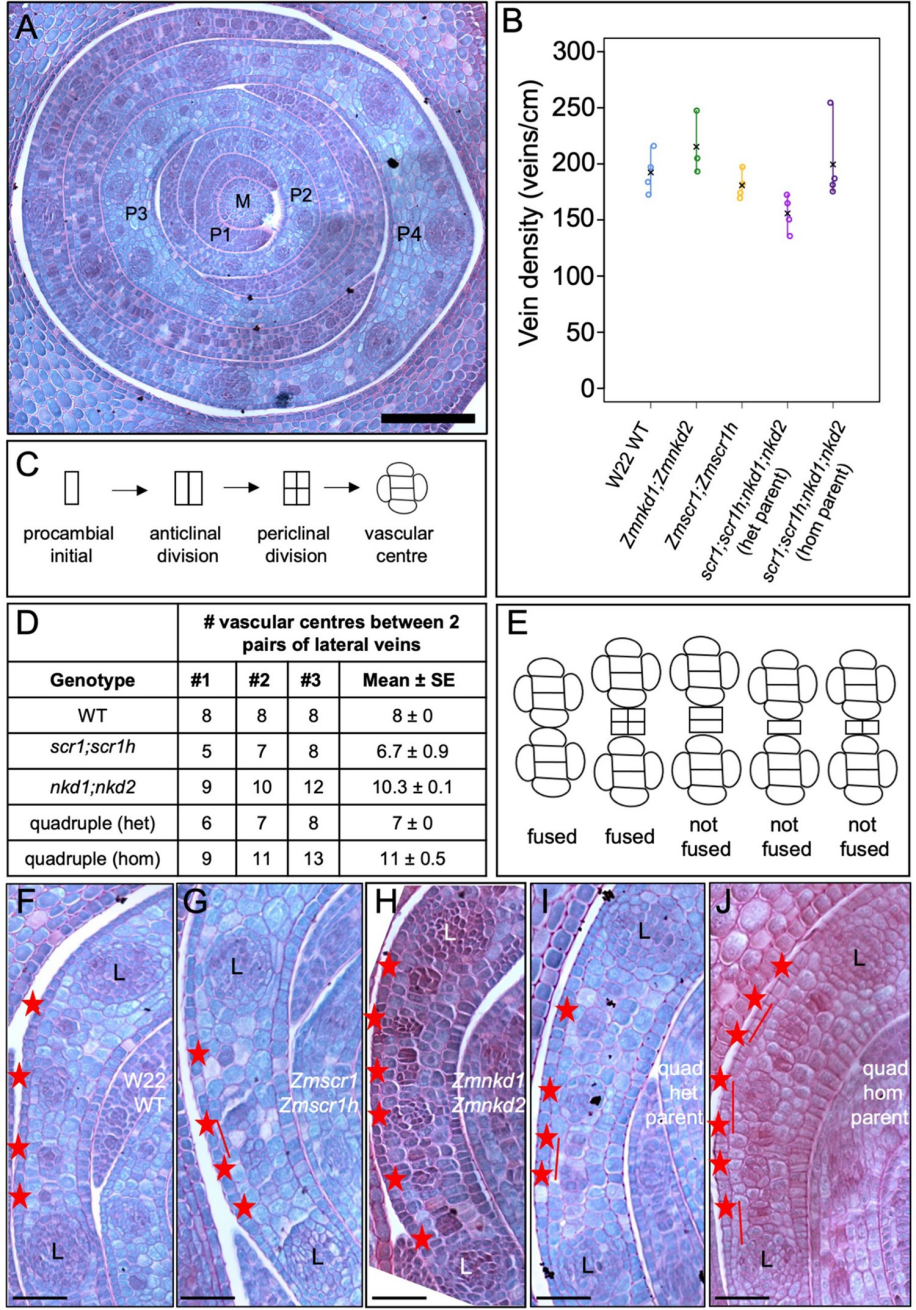
Embryonic leaf phenotypes reveal patterning defects during embryogenesis. **A)** Transverse section of a wild-type (WT) W22 embryo taken across the tip of the meristem (M), such that P1-P4 leaf primordia are visible. Scale bar: 100 μm. **B)** Quantification of vein density in P4 primordia. Open circles indicate measurements from independent biological replicates, and black crosses indicate the mean for each genotype (raw data in S1 Table). **C)** Schematic of rudimentary cell division patterns seen in the innermost ground meristem layer of the leaf as procambium is initiated to form intermediate veins. **D)** Number of vascular centres observed between two pairs of lateral veins (one each side of the midvein) in P4 primordia of three mature embryos for each genotype. Data sourced from images in S10 Fig. **E)** Schematic showing criteria used for scoring fused vein phenotype. Vascular centres were not scored as such unless both an anticlinal and periclinal division had occurred. **F-J)** Representative transverse sections of P4 primordia from WT W22 (F), *Zmscr1-m2;Zmscr1h-m1* (G), *Zmnkd1-Ds;Zmnkd2-Ds* (H), *Zmscr1-m2;Zmscr1h-m1;Zmnkd1-Ds;Zmnkd2-Ds* from a heterozygous *NKD/nkd* parent (I) and *Zmscr1-m2;Zmscr1h-m1;Zmnkd1-Ds;Zmnkd2-Ds* from a homozygous *nkd* parent (J), showing developing intermediate veins (red asterisks) between a pair of lateral (L) veins. Full images are shown in S10 Fig Scale bars: 40 μm.

When phenotyping leaves of quadruple *Zmscr1-m2;Zmscr1h-m1;Zmnkd1-Ds;Zmnkd2-Ds* mutants we noted a difference in phenotypic severity between individuals derived from different parents. Given that phenotyping was undertaken on leaf 3, which is initiated and partially patterned in the embryo, we hypothesized that the differences observed were first manifest during embryogenesis. To investigate this possibility, we sectioned through three embryos from a *Zmscr1-m2/+;Zmscr1h-m1;Zmnkd1-Ds/+;Zmnkd2-Ds/+* (*nkd* heterozygous) parent and three from a *Zmscr1-m2/+;Zmscr1h-m1;Zmnkd1-Ds;Zmnkd2-Ds* (*nkd* homozygous) parent, along with three wild-type, *Zmscr1-m2;Zmscr1h-m1* and *Zmnkd1-Ds;Zmnkd2-Ds* embryos. For quantification of veins, transverse sections were examined at the point where the tip of the meristem and P1-P4 leaf primordia (where P4 will go on to form leaf 1, P3 leaf 2 etc.) were visible (Fig 8A). Due to the anticipated challenges of identifying fused veins in young leaf primordia, where cell-division and differentiation are ongoing, we first quantified vein density in the oldest primordium to test whether it could be used as a proxy for an increase in fused veins. In most cases the oldest primordium was P4 but in one of the double *Zmscr1-m2;Zmscr1h-m1* mutants, only three primordia had been initiated. No significant difference in vein density was observed between genotypes but slightly higher densities were observed in double mutant progeny of *Zmnkd1-Ds;Zmnkd2-Ds* homozygous parents and in quadruple mutant progeny of *Zmscr1-m2/+;Zmscr1h-m1;Zmnkd1-Ds;Zmnkd2-Ds* (*nkd* homozygous) parents (Fig 8B). We next recorded the number and position of intermediate veins that had been initiated between the first two lateral veins adjacent to the midvein in the oldest primordium of each embryo. This region was selected because it is the furthest advanced developmentally and thus dividing vascular centres could be identified (Fig 8C). The midrib region was excluded because the absence of a recognizable inner ground meristem layer makes cell division patterns during procambium initiation harder to interpret. Notably a higher number of veins had been initiated in embryos derived from *Zmnkd1-Ds;Zmnkd2-Ds* double mutant parents and in quadruple mutant progeny of *Zmscr1-m2/+;Zmscr1h-m1;Zmnkd1-Ds;Zmnkd2-Ds* (*nkd* homozygous) parents (Figs 8D and S10). In the case of the quadruple mutant progeny from *nkd* homozygous parents, the number was significantly higher than in quadruple mutants from *nkd* heterozygous parents and *Zmscr1-m2;Zmscr1h-m1* double mutants (S1 Table). In two of the *Zmnkd1-Ds;Zmnkd2-Ds* double mutants, extra veins were also observed in the coleoptile, with four vascular traces present instead of the normal two (S10 Fig). This phenotype was not apparent in any other genotype. These results suggest that procambium can be initiated prematurely and/or ectopically in leaf primordia of embryos that develop in the context of a *nkd* mutant kernel.

To quantify fused veins, instances were recorded where developing vascular centres were directly adjacent to each other as opposed to being separated by one or more ground meristem cells in the innermost leaf layer. Vascular centres were classified as such if procambial initials in the innermost leaf layer had divided at least once both anticlinally and periclinally (Fig 8E). One or two instances of fused veins were observed in two of the *Zmscr1-m2;Zmscr1h-m1* double mutant embryos, a single instance was observed in two of the quadruple mutants derived from *Zmscr1-m2/+;Zmscr1h-m1;Zmnkd1-Ds/+;Zmnkd2-Ds/+* (*nkd* heterozygous) parents and at least two instances were observed in each of the three embryos derived from the *Zmscr1-m2/+;Zmscr1h-m1;Zmnkd1-Ds;Zmnkd2-Ds* (*nkd* homozygous) parent (nine events in total) (Figs 8F–8J and S10). These results suggested that loss of SCR function causes leaf patterning defects both during embryogenesis and post-germination, and raised the possibility that the enhanced patterning defects observed when combined with loss of NKD function could result as an indirect consequence of the altered kernel structure in homozygous *Zmnkd1-Ds;Zmnkd2-Ds* plants as opposed to a direct effect of loss of NKD function. Therefore, to evaluate whether NKD has a direct role, we examined leaf 6 of quadruple mutants that were derived from a *Zmscr1-m2/+;Zmscr1h-m1;Zmnkd1-Ds;Zmnkd2-Ds* parent. These leaves, which are patterned post-germination, also had an increased proportion of fused veins relative to double *Zmscr1-m2;Zmscr1h-m1* mutants (S9C–S9F Fig), demonstrating a role for NKD in leaf patterning beyond embryogenesis.

## Discussion

SCARECROW has recently emerged as a regulator of monocot leaf development [4–6,21]. Here we have demonstrated that unlike in maize and rice where SCR has distinct functions in inner leaf (maize) or stomatal (rice) patterning, in the C_4_ monocot *Setaria viridis* it undertakes both patterning functions (Figs 1 and 2). Prior to this study, no regulators that function alongside SCR in monocot leaf patterning had been identified. Here we show that *NKD IDD* genes play such a role, at least in C_4_ monocots (Fig 3). Although loss of function *nkd* mutants in maize, setaria and rice show no obvious perturbations in leaf development (Fig 4), when mutations are combined with loss of function *scr* mutations, synergistic interactions are observed in both maize and setaria, but not in rice (Figs 5–7). Most strikingly, *scr;nkd* mutants in maize and setaria exhibit an increase in the proportion of fused veins, whereby the BS cells of adjacent vascular bundles are in direct contact with no intervening M cells (Figs 6 and 7). Intriguingly, the severity of this phenotype in embryonic leaves of maize suggests that normal leaf patterning in the embryo may be perturbed by loss of *NKD* function in the endosperm (Fig 8). A summary of the leaf phenotypes of all *scr*, *nkd* and *scr;nkd* mutants examined both in this study and in previous reports is provided in Table 1. Taken together, our results provide insight into the evolution of SCR function in monocot leaves and identify another component of the patterning pathway.

**Table 1:** Summary of the leaf phenotype of maize, setaria and rice *scr*, *nkd* and *scr;nkd* mutants from this study and previous work [4,5].

Species	Mutant	Leaf phenotype
Maize	*Zmscr1;Zmscr1h*	Veins separated by only one M cell, increase in sclerenchyma and ectopic BS cells. Occasional fused veins. Stomata normal on abaxial side but slightly reduced in number on adaxial side.
	*Zmnkd1;Zmnkd2*	No obvious perturbations
	*Zmscr1;Zmscr1h;Zmnkd1;Zmnkd2*	Large increase in the number of fused veins relative to *Zmscr1;Zmscr1h* mutants. Examples of >2 veins fused together.
Setaria	*Svscr1;Svscr2*	No stomata. More veins separated by only one M cell than in wild-type
	*Svnkd*	No obvious perturbations
	*Svscr1;Svscr2;Svnkd*	Increased incidence of fused veins but fewer than in maize.
Rice	*Osscr1;Osscr2*	No stomata. No obvious inner leaf perturbations
	*Osnkd*	No obvious perturbations.
	*Osscr1;Osscr2;Osnkd*	Phenotype same as *Osscr1;Osscr2*.

We previously proposed that the role of SCR in patterning inner leaf tissues of maize may represent a C_4_-specific function, and that the stomatal role in rice may be a C_3_-specific function [5]. The analysis presented here argues against the latter because *Svscr1;Svscr2* mutants of the C_4_ species *Setaria viridis* do not develop stomata. However, inner leaf patterning perturbations were also observed in *Svscr1;Svscr2* mutants, and thus it remains plausible that the inner leaf patterning function is C_4_-specific. In this scenario, the ancestral role of SCR would be in epidermal patterning, with recruitment into Kranz patterning occurring in those monocots that evolved the C_4_ pathway. This situation contrasts with that seen in eudicots where Arabidopsis (C_3_) *scr* mutants develop normal stomata but have perturbations in BS cell identity [22,23]. On the basis of gene expression patterns and mutant phenotypes in rice, it has been proposed that SCR functions in the epidermis to interpret a positional signal (likely SHR) emanating from veins in the inner leaf [24,25]. In this way, stomatal files are correctly positioned in rows adjacent to underlying veins. It is difficult to envisage how this could happen in setaria if SCR is also expressed in the inner leaf as in maize, particularly given that the canonical model for SCR function dictates that SCR prevents SHR movement beyond the cell layer in which SCR accumulates [8,26–28]. How epidermal and inner leaf patterning processes are both mediated through the SCR pathway in setaria is a question for future research.

Another outstanding question from this study is why the severity of the fused vein phenotype is much higher in maize than in setaria *scr;nkd* mutants. In maize, many quadruple mutants exhibited more than 30% of leaf veins that were fused to an adjacent vein, whereas in setaria these incidents were rarer, and no examples were found of more than two veins fused together. One possible explanation is that because *scr;nkd* mutants in setaria have no stomata they are much weaker than those in maize. As a consequence, fewer plants survived and inner leaf phenotypes were necessarily assessed on those survivors, possibly biasing the analysis towards mutants that were less severely affected. An alternative possibility is that the mutant alleles in setaria are hypomorphic rather than null. The successful edits in *SvSCR* and *SvNKD* genes were positioned towards the 3’ end of the sequence, affecting the final 20% and 40% of the encoded proteins respectively. Whereas we can be confident that the *Svscr* alleles are null due to the conserved phenotype of *Svscr1;Svscr2* mutants with rice and maize, it is possible that the *Svnkd* alleles retain some wild-type function and that a more severe *Svscr;Svnkd* vein clustering phenotype would be observed if edits towards the 5’ end of the gene had been successful. If neither of these technical explanations is correct, the differences observed between maize and setaria must represent biological differences between the two species, which is plausible given that they are separated by millions of years of evolution and clearly deploy the SCR pathway in different developmental contexts.

IDD genes have long been known to modulate the SCR/SHR pathway in Arabidopsis. For example, JACKDAW is known to physically interact with the SCR-SHR complex [9], and it has been suggested that this interaction with an IDD C2H2 transcription factor partner enables SCR-SHR to bind DNA and activate expression of downstream targets [29]. There are IDD orthologs in monocot genomes, but very little functional insight into their roles. This may be in part due to the extensive functional redundancy in grass genomes, which combined with the challenges of generating loss-of-function lines in monocots makes functional analysis challenging. In support of this suggestion, even in Arabidopsis where many IDD mutants have been characterized, phenotypic perturbations are often only observed when higher order mutants are examined [11]. This observation fits with our finding that a role for NKD in leaf patterning in maize and setaria is only revealed when *NKD* genes are mutated alongside *SCR* genes. The lack of phenotypic perturbations in leaves of *Zmnkd1;Zmnkd2* mutants may be explained by a direct or indirect feedback loop between NKD and SCR (Fig 3A). In leaves of *Zmnkd1;Zmnkd2* mutants, both *ZmSCR1* and *ZmSCR1h* transcripts accumulate at approximately two-fold higher levels than normal (Fig 3A), which may at least partially compensate for loss of NKD function. Conversely, *ZmNKD1* and *ZmNKD2* transcripts accumulate at lower levels than normal in *Zmscr1;Zmscr1h* mutants (Fig 3A), creating a scenario that more closely resembles the quadruple mutant. Despite the paucity of studies examining IDD gene function in monocots, it was recently shown that IDD12 and IDD13 act alongside SHR in rice to regulate ground meristem proliferation in leaves [30]. This finding is intriguing because we find here that *NKD* plays a similar role alongside *SCR* in C_4_ inner leaf patterning, but that neither gene functions in the inner leaf of rice (Fig 6) [5]. It may be that different IDD genes fine-tune the action of both SCR and SHR by controlling the activation of distinct downstream targets in different developmental contexts.

The accumulation of *SCR* and *NKD* transcripts in the ground meristem cells between developing veins, and the fused veins observed in both maize and setaria *scr;nkd* mutants imply two possible mechanisms for SCR and NKD function. The first is that the combined action of SCR and NKD promotes cell division in the ground meristem and then specifies those cells as mesophyll. In this model, in the absence of SCR and NKD ground meristem cells do not divide and/or are mis-specified such that veins develop in direct contact with each other. This would be analogous to the role played by SCR in the arabidopsis root, where loss of gene function leads to loss and mis-specification of the endodermal cell-layer [7]. The second is that SCR and NKD act to inhibit the formation of veins in regions between existing veins. In their absence, veins are formed in regions between already specified veins, resulting in the runs of fused veins observed in maize and setaria *scr;nkd* mutants. This mechanism may be analogous to the lateral inhibition mechanism that patterns trichomes and stomata on the leaf surface [31–33], whereby the fused veins represent a clustering phenotype caused by absence of the inhibitor. Because SCR and NKD do not accumulate in developing veins, in this scenario it is likely that they would interpret an inhibitory signal emanating from adjacent specified veins. In either case, the inner leaf perturbations observed in *scr* but not *nkd* mutants suggest that SCR can compensate for loss of NKD function in the inner leaf patterning pathway (either though a transcriptional feedback loop or via alternative pathways) whereas NKD cannot compensate for loss of SCR function, a relationship that is similar to that seen in other pathways where SCR and IDD proteins interact [9–11]. An alternative hypothesis is that an unidentified third factor acts redundantly with both SCR and NKD. In this scenario, the unidentified factor would fully compensate for loss of NKD function but only partially compensate for loss of SCR function, explaining the normal phenotype of *nkd* mutant leaves and why some veins develop normally even in *scr;nkd* quadruple mutants.

NKD has a well-defined role in the endosperm of maize, where it acts to suppress proliferation of the aleurone layer–the aleurone is a single cell layer in wild-type endosperm whereas in *nkd1;nkd2* mutants it is multi-layered [17]. Within the endosperm, *NKD1* and *NKD2* are expressed in both the aleurone and the starchy endosperm, and loss of function of either gene is compensated for by upregulation of the other [17]. Although low levels of *NKD* gene expression have also been reported in the developing embryo [17], the data reported here suggest that loss of *NKD* function in the endosperm may perturb the patterning of leaves in the embryo. Without further experimentation it is impossible to determine the mechanistic basis of this presumably non cell-autonomous effect. Perturbations to leaf patterning in embryos developing within *nkd1;nkd2* mutant seed could either be an indirect consequence of disrupted signalling through the mutant aleurone layer or a direct consequence of loss of activity of NKD and/or its downstream targets. Speculation at this stage would be premature because reciprocal crosses are needed to determine whether there is a direct (maternal) effect. Taken together, the data presented here suggest that NKD is a regulator of leaf patterning in C_4_ grasses, acting with SCR to promote M cell specification or repress vein specification, and possibly influencing the patterning of embryonic leaves in a non-cell autonomous manner.

## Materials and methods

### Phylogenetics

Primary transcript proteomes from Zea mays (B73), Sorghum bicolor, Setaria italica, Setaria viridis, Oryza sativa, Brachypodium distachyon, Brachypodium stacei, Ananas comosus, Arabidopsis thaliana, Solanum lycopersicum and Physcomitrella patens were downloaded from Phytozome12 [34]. The ZmNKD1 (GRMZM2G129261) primary protein sequence was used as a query in a BLASTp search (e-value of 1e-3) against these proteomes. The top 100 results were aligned using MAFFT-linsi [35] and the alignment was then used to generate a maximum likelihood phylogeny using IQtree [36,37].

### Plant material and growth conditions

Maize inbred line W22 and *Zmnkd1-Ds;Zmnkd2-Ds* (introgressed into W22) seed were obtained from Phil Becraft, Iowa State University. *Zmscr1-m2;Zmscr1h-m1* seeds were generated and reported previously [4]. W22 was used as the wild-type control in all maize experiments except for *in situ* hybridization experiments where the inbred line B73 was used. *Setaria viridis* accession ME034V was used as the wild-type control for all setaria experiments and *Oryza sativa spp japonica* cv. Kitaake was used as the wild-type control for all rice experiments. *Osscr1;Osscr2* seed were generated and reported previously [5]

Maize and setaria plants for phenotypic analysis were grown in a greenhouse in Oxford, UK with 16 h light/ 8 h dark cycle, daytime temperature 28°C and night-time temperature 20°C, with supplemental light provided when natural light levels were below 120 μmol photon m^−2^ s^−1^[4]. Maize seed were germinated and grown as described previously [4]. Setaria seed were treated for at least 3 weeks in damp sphagnum moss (Zoo-Med Laboratories Inc) at 4°C prior to germination to break dormancy. Seed were then germinated on damp paper towels in sealed petri dishes, in a growth cabinet with the same conditions as the greenhouse. After seven days, seedlings were transferred to Sinclair compost in 60 well modular trays for growth in the greenhouse. Plants grown for seed propagation were re-potted after 4 weeks into 7.5cm pots with the same compost, for growth to maturity. Rice seed were sterilised prior to germination on ½ MS media and subsequently transferred to a hydroponic growth system in 50 ml falcon tubes, as described previously [5].

### Mutant nomenclature

Maize, setaria and rice mutant alleles are indicated by the suffixes *Zm*, *Sv* and *Os* respectively. *SCR* and *NKD* mutant alleles are indicated in lower-case italics, with specific alleles indicated by a dash and the allele reference (e.g. *Zmscr1-m2*). Higher order mutants are indicated by individual alleles separated by a semi-colon, and plants heterozygous for a specific allele are indicated by a forward slash followed by the second allele, or a plus indicating the wild-type allele (e.g. *Zmscr1-m2/+;Zmscr1h-m1*).

### Generation of maize quadruple mutant and genotyping

Pollen from *Zmscr1-m2/+;Zmscr1h-m1* plants (double mutants do not produce pollen or ears) was used to cross to *Zmnkd1-Ds;Zmnkd2-Ds* homozygous ears. 50% of the F1 plants were thus heterozygous for all four mutant alleles, and these plants were self-pollinated. In the subsequent F2, kernels with the shrunken *nkd1/nkd2* phenotype were selected for self-pollination. F3 seed derived from individuals with *Zmscr1/+;Zmscr1h;Zmnkd1;Zmnkd2* or *Zmscr1;Zmscr1h/+;Zmnkd1;Zmnkd2* genotypes were then used for quadruple mutant analysis.

Genomic DNA for genotyping was extracted either using a modified SDS 96-well plate method or a CTAB method depending on the number of samples to be processed, as described previously [4]. PCR genotyping assays were used to track the genotype at each locus through the generations. The *Zmscr1-m2* and *Zmscr1h-m1* alleles were genotyped as described previously [4]. The expected *Ds* insertion site in the *Zmnkd1-Ds* allele was confirmed by two separate PCR assays, one using two primers (nkd1-F, TATCTTATCCGTCGATGCGTTG and nkd1-R, TCGGTCATGGCATCCTGCCTCCG) that flanked the insertion site and produced an amplicon for the wild-type *ZmNKD1* sequence, and another using one flanking primer (nkd1-F) and a second primer nested within the Ds transposon sequence (W22-Ds-R1, GGAGCTGGCCATATTGCAGTCATC) that produced an amplicon when the *Zmnkd1-Ds* sequence was present. The *Zmnkd2-Ds* allele referred to here was previously described as *nkd2-Ds0766* and believed, like *Zmnkd1-Ds*, to be a loss-of-function allele caused by the insertion of a *Ds* transposon. However, attempts to amplify from the *Ds* transposon sequence to the region flanking the insertion failed. Instead, we demonstrated that loss-of-function of this allele was due to a deletion at the 5’ end of the gene, with amplification (primers ZmNKD2-F2, CTTGTTGCCGTTGTTGATTG and ZmNKD2-R2, GTGCCATGTGGCTCCTATTT) yielding a fragment that was 786bp smaller in homozygous *Zmnkd2-Ds* plants than in wild-type (Figs 4A and S11). This deletion is believed to be caused by a chromosomal rearrangement (Phil Becraft, personal communication). As expected, *Zmnkd1-Ds;Zmnkd2-Ds* seed always exhibited the shrunken kernel phenotype characteristic of *Zmnkd1;Zmnkd2* double mutants. PCR amplifications were undertaken using GoTaq DNA polymerase (Promega) and PCR cycles of 95°C for 5 min; 35 cycles of 95°C for 30 s, 57–64°C for 30 s and 72°C for 60–90 s; and 72°C for 5 min. Betaine (1 M; Sigma Aldrich) was added to all reactions to aid amplification of regions with high-GC content.

### Generation of rice and setaria CRISPR lines

Rice and setaria *NKD* orthologs were identified from the phylogeny presented in Fig 3. Setaria *SCR* orthologs were identified from a previously published phylogeny [4]. The *OsNKD* sequence was obtained from phytozome V12 and two guide RNAs targeting the 5’ end of the *c*oding sequence were designed using CRISPOR [38] (OsNKD-g59: CTTCGGGATTAGCAGGGATG, OsNKD-g72: GTGATATCAGCGGCTTAATC). Guide RNAs targeting *OsSCR1* and *OsSCR2* were designed and used previously [5], and two of these (OsSCR1-g397: TCCACCCAAGCCGTACTAGG, OsSCR2-g507: CGAGGTCGGGGTTACATGGC) were used in this study. Guides were cloned as described previously into four constructs targeting either *OsNKD* (EC17821: OsNKD-g59 and EC17822: OsNKD-g72) or *OsSCR1*, *OsSCR2* and *OsNKD* (EC17827: OsSCR1-g397, OsSCR2-g507, OsNKD-g59 and EC17828: OsSCR1-g397, OsSCR2-g507, OsNKD-g72). *SvSCR1*, *SvSCR2* and *SvNKD* sequences were obtained from phytozome V12, however, as the ME034V accession used for transformation is not the same as the sequenced accession, we first amplified the target regions of the ME034V gene sequences. Surprisingly, we found that the ME034V *SvSCR1* and *SvSCR2* sequences more closely matched the published *Setaria italica* sequences, and the ME034V *SvNKD* sequence was intermediate between the published *viridis* and *italica* sequences. Confirmed ME034V sequences were used in subsequent guide RNA design.

Due to a low editing efficiency of guide RNAs in *Setaria viridis* (Daniela Vlad, personal communication), multiple guides were designed against each gene (S1 Fig). Because *SvSCR1* and *SvSCR2* have high sequence similarity, a common set of six guides were designed to target both genes simultaneously. For both *SvSCR* and *SvNKD*, guides were positioned towards the 5’ end of the gene to make complete loss-of-function alleles more likely. However, at least one guide was positioned closer to the 3’ end of the gene both to increase the chance of obtaining mutations and to enable the potential excision of most of the gene sequence. To minimise the number of constructs for transformation, guides were cloned into polycistronic expression modules separated by tRNA spacers [39]. Two such polycistronic guide arrays were assembled, one with the six *SvSCR* guides and one with the four *SvNKD* guides (S1 Fig), both driven by the rice U3 promoter. These arrays were then combined into three constructs using the same Golden Gate cloning system described previously for rice [5]: C402052 (SvSCR array), C402053 (SvNKD array) and C402054 (SvSCR and SvNKD arrays). Three identical constructs were cloned that included the GRF-GIF1 fusion previously shown to enhance rice and wheat transformation efficiency [40]. No enhancement in setaria transformation was observed here, however, one line that was generated from one of these constructs (C402057: SvSCR and SvNKD arrays) was used for subsequent phenotypic analysis after the construct (and thus GRF-GIF1 fusion) were segregated away from the mutations.

Constructs were transformed into Kitaake rice seed using agrobacterium strain EHA105 as described previously [5]. Constructs were transformed into setaria ME034V using a modified transformation protocol [41]. In brief, ME034V seed were dehulled and sterilised with 10% bleach plus 0.1% tween for 3 minutes. Seed were placed on callus induction media (CIM) (4.3 g/L MS salts, 40 g maltose, 35 mg/L ZnSO_4_^.^7H2O, 0.6 mg/L CuSO_4_^.^5H_2_0, 0.5 mg/L kinetin, 2 mg/L 2,4-D 4 g/L Gelzan, pH 5.8) and after 4–5 weeks embryonic callus was moved to fresh media and gelatinous callus removed. After a further 3 weeks, gelatinous callus was again removed and the remaining callus moved to fresh media for 1–2 weeks prior to transformation. Constructs were cloned into agrobacterium strain AGL1 and grown in liquid media to an OD of ~0.6. Agrobacteria were then collected by centrifugation and resuspended in 50ml liquid CIM media with the addition of 40 μm acetosynringone and 0.02% synperonic acid. Around 100 pieces of calli were added to this suspension and incubated for 5 minutes with occasional rocking prior to drying and transfer to fresh CIM plates with filter paper placed on top of the media. Calli were co-cultivated for 3 days at 22°C before transfer to CIM selective plates (CIM containing 150 mg/L timentin and 40mg/L hygromycin for 16 days at 24°C in the dark. Calli were then transferred to plant regeneration media (PRM) (4.3 g/L MS salts, 20 g/L sucrose, 7 g/L Phytoblend, 2 mg/L kinetin, 150 mg/L timentin, 15 mg/L hygromycin, pH 5.8) and moved to fresh media every 2 weeks. Emerging shoots were dissected from calli and moved to rooting media (RM) (2.15 g/L MS salts, 30 g/L sucrose, 7 g/L Phytoblend, 150 mg/L timentin, 20 mg/L hygromycin, pH 5.7). Shoots that survived this stage were transferred to compost for genotyping.

Genomic DNA for rice and setaria T0 genotyping was obtained using the same methods described for maize. T0 seedlings were first screened using primers that amplified a fragment of the hygromycin gene [5] to assess transformation success. Gene specific primers were then used to amplify and sequence the region targeted for editing. In rice, all guides induced successful edits, however, in the case of construct EC17822 only a few transformed plants were obtained and thus edited plants from EC17821 were taken forward for *Osnkd* single mutant characterization. In setaria, only one guide for both *SCR* and *NKD* (SvSCR-ex2g49: GAGCAGGACCTGAGCCACTC and SvNKD-ex3g438: CATGAGTCCATGGAACGGCT) was found to consistently yield successful edits. In both cases this guide was positioned closer to the 3’ end of the gene. In the case of *SCR*, out-of-frame mutations created by the successful guide resulted in the final ~20% of the protein sequence being either nonsense or prematurely truncated by an early stop codon, whereas for *NKD* the final ~40% of the protein was affected. As was the case when generating rice *scr* mutants [5], no plants were identified in the T0 generation that had out-of-frame mutations in all four *SCR* alleles, with all screened plants having at least one unedited copy of one of the *SCR* genes. Interestingly, some T0 plants exhibited pale sectors in leaves, but only in plants transformed with the *SCR* guide array. All *Svnkd* mutations corresponded to the same C deletion (*Svnkd-m1*), and plants homozygous for this edit were viable. For both rice and setaria, T1 lines were screened to identify mutated plants that had segregated away from the construct. From this analysis, two independent *Osnkd* lines, three *Osscr1;Osscr2;Osnkd* lines (alongside two previously generated *Osscr1;Osscr2* lines), two *Svscr1;Svscr2* lines, two *Svnkd* lines and three *Svscr1;Svscr2;Svnkd* lines were prioritized for phenotypic characterization in the T2 and T3 generation.

### Leaf phenotyping

Fully expanded leaves were used to obtain transverse leaf sections of maize, setaria and rice. For maize and setaria a region from the midpoint along the proximal-distal axis was cut and placed in molten 7% agar. Once solidified, blocks were trimmed down and mounted using superglue for sectioning on a vibratome. Sections of 30–60 μm were cleared in either 3:1 ethanol: acetic acid (maize) or 70% ethanol (setaria) for 10 minutes. *Svscr1;Svscr2* and *Svscr1;Svscr2;Svnkd* leaves were sufficiently pale for leaf anatomy to be visible without any clearing. Sections were transferred to slides and imaged using a Leica DMRB microscope with a DFC7000T camera under either brightfield or UV illumination using Leica LASX image analysis software. Rice leaf transverse sections were obtained by first fixing a segment from the midpoint along the proximal-distal axis of fully expanded leaf 5 in 3:1 ethanol: acetic acid for 30 mins, before transferring to 70% ethanol. Samples were wax infiltrated, sectioned at 10 μm using a Leica RM2135 rotary microtome and stained with Safranin O and Fastgreen as described previously [5]. Stomatal impressions of setaria leaves were obtained and imaged under phase-contrast microscopy using the same dental resin and nail varnish method described previously [5].

### Seed phenotyping

Setaria seed were dehulled and imbibed for 48 hours in water before being fixed in FAA (4% formaldehyde, 5% acetic acid, 50% ethanol) for 2 months prior to sectioning. Seed were then embedded in paraffin as described previously [5] and sectioned into 20μm sections using a Leica RM2135 rotary microtome. The resultant sections were incubated in Histoclear (2 x 10 min), and then re-hydrated through 100%, 95%, 90%, 80%, 70%, 50%, 30% and 10% (w/v) ethanol, all at room temperature. Sections were then rinsed in distilled H_2_O, stained for 5 seconds in 0.05% (w/v) Toluidine Blue (50 mM citrate buffer, pH 4.4), and then rinsed in distilled H_2_O again, before finally being mounted using a drop of entellen (Merck Millipore). Slides were imaged under brightfield using the same microscope described above. Rice and setaria whole seed were photographed using a Leica S9i stereo microscope.

### *In situ* hybridization

*In situ* hybridization was carried out using wax-embedded shoot apices as described by [25], with digoxygenin (DIG)-labelled RNA probes designed to specifically detect either *ZmSCR1* or *ZmNKD1* transcripts. The *ZmSCR1* probe was a 108 bp region towards the end of the first exon, which shared 78% identity with the corresponding region of *ZmSCR1h* [4]. The *ZmNKD1* probe was a 116 bp region of the 5’UTR ending 12 nucleotides upstream of the ATG. The probe shared 59% identity with the corresponding region of *ZmNKD2* and with the stringency conditions used was expected to be specific for *ZmNKD1*. Post-hybridization washes were undertaken with 0.005 x SSC buffer made from a 20x SSC stock (3M NaCl, 0.3M Na_3_citrate), calculated to ensure stringency.

### Maize kernel genotyping and embryo imaging

The endosperm of kernels from segregating seed packets was first chipped and used for genotyping using the CTAB DNA extraction method described above, except samples were homogenized prior to the addition of extraction buffer. The resultant genomic DNA was used as template in PCR amplifications to identify homozygous mutants for phenotypic characterization. Kernels of interest were then soaked overnight in water, to enable the removal of the embryo the following day. Embryos were fixed in FAA (10% formaldehyde, 5% acetic acid, 50% ethanol) and vacuum infiltrated before incubation overnight in fresh FAA. Samples were transferred to 50% and then 70% ethanol the next day, and wax infiltrated as described previously [5]. 10 μm transverse sections were obtained using a microtome just beneath the tip of the meristem where all primordia are visible, and stained with Safranin O and Fastgreen as described above. Slides were imaged using brightfield illumination and the whole region of interest obtained using the Leica XY builder software.

### qPCR

RNA was extracted using the RNeasy kit (Qiagen) from whole maize shoots of both *Zmscr1-m2;Zmscr1-m1* and *Zmnkd1-Ds;Zmnkd2-Ds* mutants and the respective wild-type lines, 7 days after germination. RNA was DNase treated (TURBO DNase, Thermo Fisher) and used as template to generate cDNA (Maxima first strand cDNA synthesis kit, Thermo Fisher). Primers were designed to amplify short fragments of *ZmSCR1*, *ZmSCR1h*, *ZmNKD1* and *ZmNKD2* gene sequences as well as two housekeeping genes (*ZmCYP* and *ZmEF1α*) verified previously as suitable for use in maize qRT-PCR (S3A Fig) [42]. RT-PCR was used to verify that a single product of the correct size was amplified. Quantitative RT-PCR amplifications were undertaken using SYBR-Green (Thermo Fisher) with cycling conditions 95°C for 10 min, then 40 cycles of 95°C for 15 s and 60°C for 1 min. Melt curves were obtained by heating the resultant product from 60°C to 95°C to confirm that a single product was amplified for each primer pair (S3B Fig). Amplification efficiencies were assessed using a dilution series of cDNA template with >80% taken to be suitable. In all cases three technical replicates with Ct range <0.5 were obtained from each sample and all comparisons were run on the same 96-well plate. Ct values were obtained using the qPCR miner algorithm [43] and fold-change between wild-type and mutants was calculated using the 2−^ΔΔCT^ method [44]. A combined wild-type average was used to compare each individual wild-type to indicate the range of the wild-type data. Mutant samples were then compared with the same overall wild-type average. Values <1 indicate a relative reduction compared with wild-type whereas values >1 indicate a relative increase compared to wild-type.

### Statistical analyses

Statistical tests were undertaken using R Studio. Welch’s *t*-test (two-tailed) were performed for analyses involving only two groups. One-way ANOVA were performed for analyses involving more than two groups, followed by Tukey’s HSD post-hoc testing if the ANOVA *p* value was <0.05. Fold-change data was log-transformed prior to analysis. Details of all statistical analyses are shown in S1 Table.

## Supporting information

S1 Fig Summary of setaria CRISPR design. **A)** Cartoon summaries of *SvSCR1/2* and *SvNKD* genes with guide positions indicated by black arrows above each gene sequence. 5’ and 3’ untranslated regions are depicted in purple, coding sequences in green and introns in orange. **B)** Guide RNA names and sequences. Guides that successfully edited are highlighted green. **C)** Cartoon summary of Level 1 and Level 2 Golden Gate constructs designed for this study. Promoters are indicated by arrows, and expression modules by rectangles. **D)** Genotyping primers used in this study.
(TIF)

S2 Fig *Svscr1* and *Svscr2* single mutants are phenotypically indistinguishable from wild-type. **A-E)** Images of wild-type (WT) ME034V (A), *Svscr1-m1* (B), *Svscr1-m2* (C), *Svscr2-m1* (D) and *Svscr2-m2* (E) plants taken 21 days after sowing. Scale bars: 10cm.
(TIF)

S3 Fig Primers used for quantitative RT-PCR. **A)** Primer sequences used for quantitative RT-PCR of each gene in this study. **B)** Melt-curves for each primer pair.
(TIF)

S4 Fig Summary of rice CRISPR design. **A)** Cartoon depiction of *OsNKD* with guide positions indicated above the gene with black arrows. 5’ and 3’ untranslated regions are depicted in purple, coding sequences in green and introns in orange. **B)** Guide RNA sequences used in this study. **C)** Cartoon summary of Level 2 Golden Gate constructs used to generate *Osnkd* (17821) and *Osscr1;Osscr2;Osnkd* (17827 and 17828) mutants. **D)** Primer sequences and restriction digests used to genotype *OsNKD* editing events.
(TIF)

S5 Fig *nkd* mutants do not have perturbed seed development in setaria or rice. **A-D)** Images of setaria (A & B) and rice (C & D) seed with (A & C) or without (B & D) the husk for both wild-type (WT) (top rows) and *nkd* mutants (bottom rows). **E-G)** Cross sections of setaria WT ME034V (E), *Svnkd-m1* (line 1) (F) and *Svnkd-m1* (line 2) (G) mature seed. The aleurone layer is indicated by Al in each image.
(TIF)

S6 Fig *Osnkd* and *Svnkd* mutants do not exhibit perturbed leaf development. **A-D)** Stomatal impressions of the abaxial surface of wild-type (WT) Kitaake rice (A), *Osnkd-m6* (B), WT setaria ME034V (C) and *Svnkd-m1* (D) leaves. Rice images are taken of leaf 5 and setaria images of leaf 3. Stomata are false coloured orange. Scale bars: 100 μm. **E, F)** Cross sections of WT Kitaake (E) and *Osnkd-m6* (F) leaf 5 taken from the midpoint along the proximal-distal axis. Scale bars: 200 μm.
(TIF)

S7 Fig *Svscr1;Svscr2;Svnkd* mutant leaves exhibit occasional fused veins with no intervening mesophyll cells. Quantification of the number of M cells in five setaria genotypes: wild-type ME034V, *Svscr1-m1;Svscr2-m3*, *Svscr1-m2;Svscr2-m1*, *Svscr1-m4;Svscr2-m1;Svnkd-m1* and *Svscr1-m1;Svscr2-m2;Svnkd-m1*. Quantification undertaken on leaf 4. Bars are the standard error of the mean. Biological replicates (n =) are indicated and letters in the top right corner of each plot indicate statistically different groups (*P*≤0.05, one-way ANOVA and Tukey’s HSD) calculated using the mean number of M cells in each genotype (raw data in S1 Table).
(TIF)

S8 Fig Examples of fused veins in *Svscr1-m1;Svscr2-m2;Svnkd-m1* plants. **A-B)** Transverse sections of two additional *Svscr1-m1;Svscr2-m2;Svnkd-m1* mutant leaves, taken at the midpoint along the proximal-distal axis of leaf 4, imaged under UV illumination. White boxes indicate examples of fused veins. Scale bars are 50 μm.
(TIF)

S9 Fig Quadruple *Zmscr1-m2;Zmscr1h-m1;Zmnkd1-Ds;Zmnkd2-Ds* mutants exhibit increased fused leaf veins in a variety of contexts. **A)** Quantification of the percentage of fused veins in leaf 3 of double *Zmscr1-m2;Zmscr1h-m1* mutants pre- and post-outcross to *Zmnkd1-Ds;Zmnkd2-Ds*. Mutants phenotyped post-outcross were also segregating for *Zmnkd1-Ds* and *Zmnkd2-Ds* (genotypes labelled on figure). **B)** Quantification of the percentage of fused veins in leaf 3 of wild-type (WT) W22, *Zmscr1-m2;Zmscr1h-m1* and two additional quadruple mutants from two outcrosses using independent *Zmnkd1-Ds;Zmnkd2-Ds* plants. Quadruple mutants from both outcrosses were segregating in progeny of selfed *Zmscr1-m2/+;Zmscr1h-m1;Zmnkd1-Ds;Zmnkd2-Ds* parents. **C-E)** Transverse sections of leaf 6 from WT W22, *Zmscr1-m2;Zmscr1h-m1* and *Zmscr1-m2;Zmscr1h-m1;Zmnkd1-Ds;Zmnkd2-Ds* lines. In (E) fused veins are indicated by arrows. Scale bars: 100 μm. **F)** Quantification of the percentage of fused veins in WT W22, *Zmscr1-m2;Zmscr1h-m1* and *Zmscr1-m2;Zmscr1h-m1;Zmnkd1-Ds;Zmnkd2-Ds* leaf 6. In (A), (B) and (F) open circles are data points from biological replicates, and black crosses indicate the mean for each genotype. Letters above each genotype indicate statistically different groups (*P*≤0.05, one-way ANOVA and Tukey’s HSD (raw data in S1 Table).
(TIF)

S10 Fig Leaf patterning defects are evident in mature embryos of maize. **A-C)** Cross sections of mature embryos of wild-type W22 (A), double *Zmscr1-m2;Zmscr1h-m1* mutants (B) and double *Zmnkd1-Ds;Zmnkd2-Ds* mutants (C). Double *Zmscr1-m2;Zmscr1h-m1* mutants were derived from selfed *Zmscr1-m2/+;Zmscr1h-m1* parents whereas double *Zmnkd1-Ds;Zmnkd2-Ds* mutants were derived from selfed double mutant parents. Three embryos of each genotype are shown. Lateral (L) and developing intermediate (V or V* if very early in development) veins are indicated in the oldest leaf primordium, which in most cases is at plastochron (P) 4. Instances of fused veins are indicated by red lines. **D)** Cross sections of the double *Zmnkd1-Ds;Zmnkd2-Ds* mutant embryos in (C) shown at lower magnification to illustrate coleoptile phenotypes. Arrows point to vascular centres in the coleoptile. **E, F)** Quadruple mutants derived from selfed *Zmscr1-m2/+;Zmscr1h-m1;Zmnkd1-Ds/+;Zmnkd2-Ds/+* (*nkd* heterozygous) (E) or selfed *Zmscr1-m2/+;Zmscr1h-m1;Zmnkd1-Ds;Zmnkd2-Ds* (*nkd* homozygous) (F) parents. Labels as for (A-C).
(PDF)

S11 Fig *Zmnkd2-Ds* genotyping. Genotyping of the *Zmnkd2-Ds* allele revealing a 786bp deletion.
(TIF)

S1 Table Summary of statistical analyses. (XLSX)
