# Supplementary figures and images for: Mutations in NAKED-ENDOSPERM IDD genes reveal functional interactions with SCARECROW during leaf patterning in C4 grasses
Source: PLoS Genet. 2023 Apr 17;19(4):e1010715. doi: 10.1371/journal.pgen.1010715 (PMC10138192; doi:10.1371/journal.pgen.1010715)

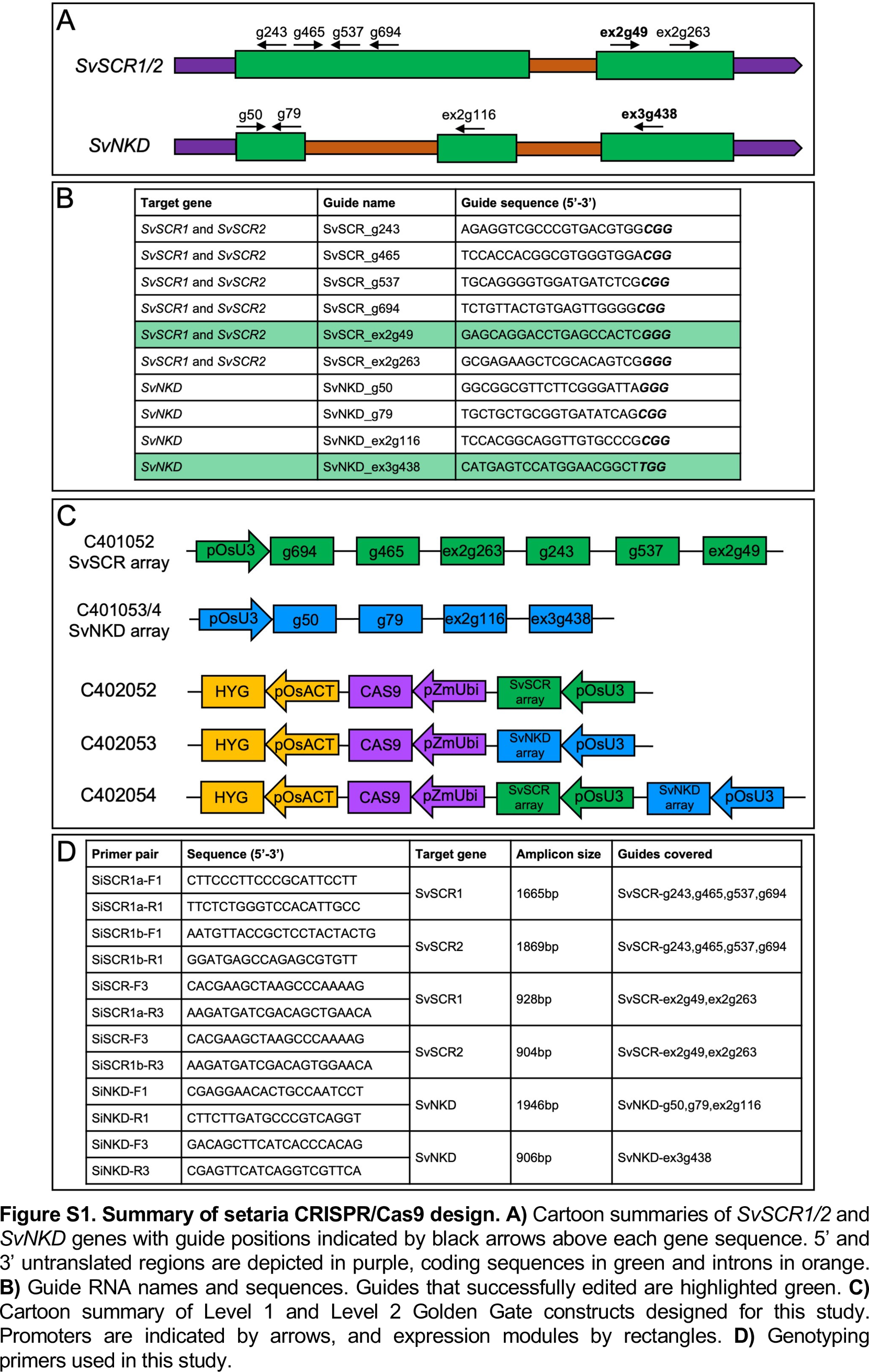

Supplement: S1 Fig — A) Cartoon summaries of SvSCR1/2 and SvNKD genes with guide positions indicated by black arrows above each gene sequence. 5’ and 3’ untranslated regions are depicted in purple, coding sequences in green and introns in orange. B) Guide RNA names and sequences. Guides that successfully edited are highlighted green. C) Cartoon summary of Level 1 and Level 2 Golden Gate constructs designed for this study. Promoters are indicated by arrows, and expression modules by rectangles. D) Genotyping primers used in this study. (TIF) [file pgen.1010715.s001.tif]

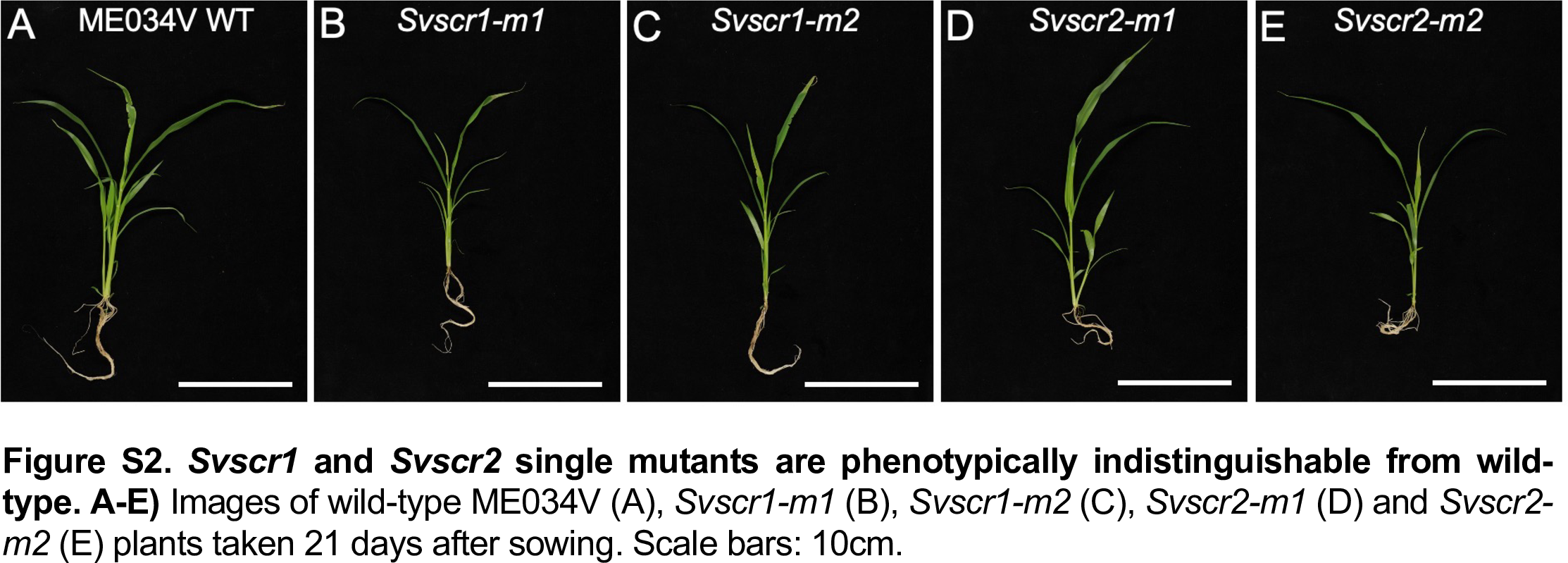

Supplement: S2 Fig — A-E) Images of wild-type (WT) ME034V (A), Svscr1-m1 (B), Svscr1-m2 (C), Svscr2-m1 (D) and Svscr2-m2 (E) plants taken 21 days after sowing. Scale bars: 10cm. (TIF) [file pgen.1010715.s002.tif]

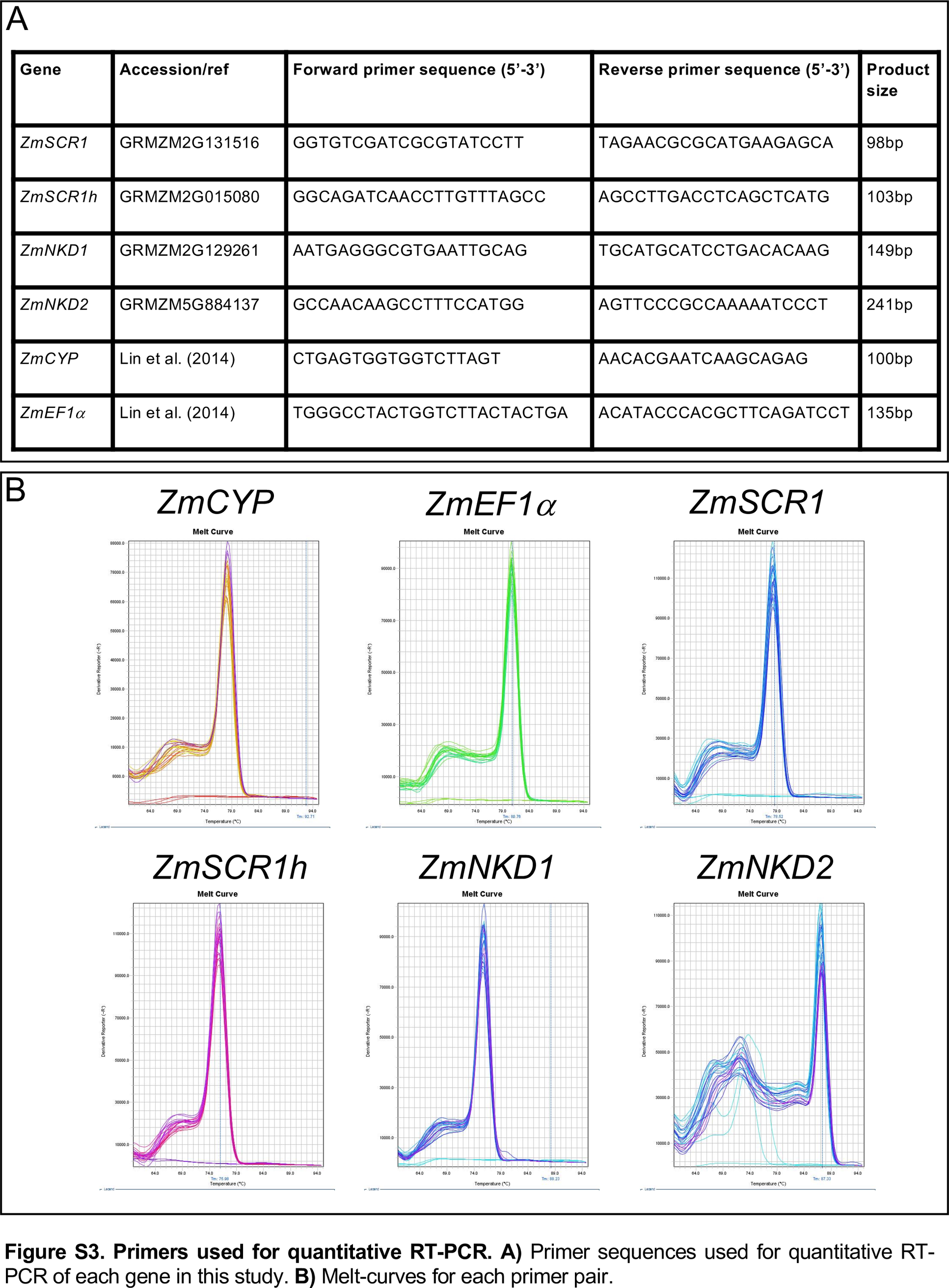

Supplement: S3 Fig — A) Primer sequences used for quantitative RT-PCR of each gene in this study. B) Melt-curves for each primer pair. (TIF) [file pgen.1010715.s003.tif]

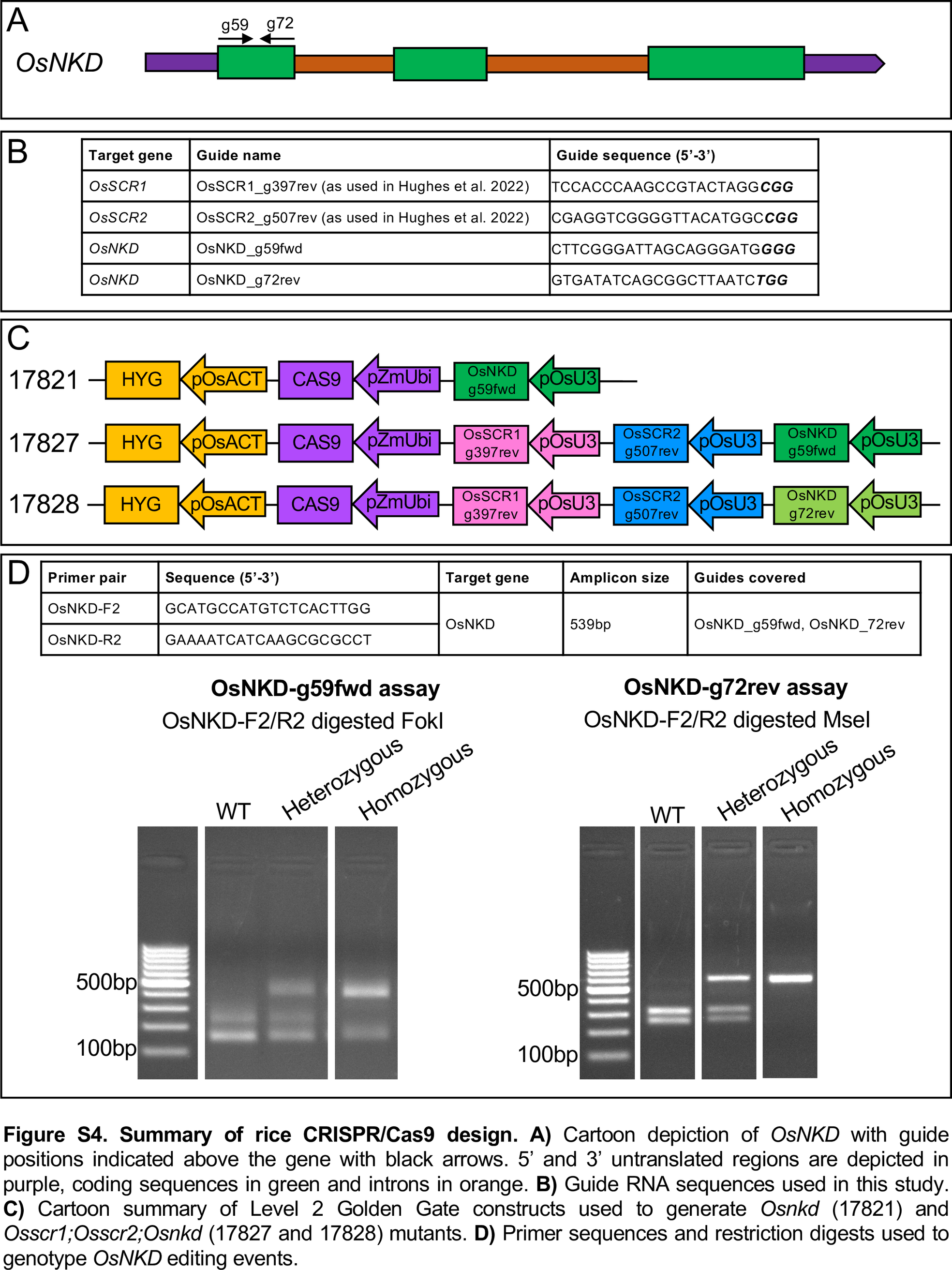

Supplement: S4 Fig — A) Cartoon depiction of OsNKD with guide positions indicated above the gene with black arrows. 5’ and 3’ untranslated regions are depicted in purple, coding sequences in green and introns in orange. B) Guide RNA sequences used in this study. C) Cartoon summary of Level 2 Golden Gate constructs used to generate Osnkd (17821) and Osscr1;Osscr2;Osnkd (17827 and 17828) mutants. D) Primer sequences and restriction digests used to genotype OsNKD editing events. (TIF) [file pgen.1010715.s004.tif]

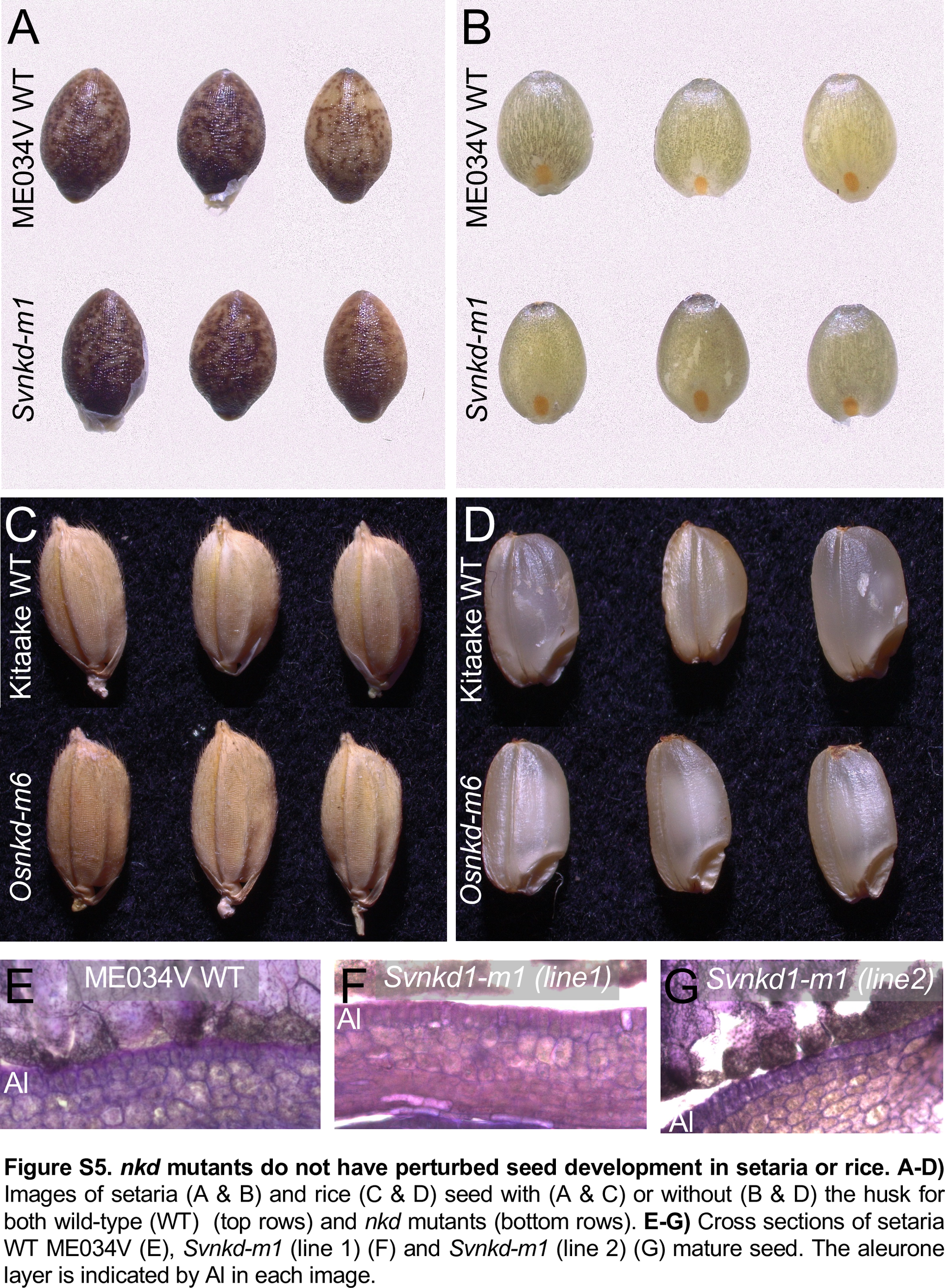

Supplement: S5 Fig — A-D) Images of setaria (A & B) and rice (C & D) seed with (A & C) or without (B & D) the husk for both wild-type (WT) (top rows) and nkd mutants (bottom rows). E-G) Cross sections of setaria WT ME034V (E), Svnkd-m1 (line 1) (F) and Svnkd-m1 (line 2) (G) mature seed. The aleurone layer is indicated by Al in each image. (TIF) [file pgen.1010715.s005.tif]

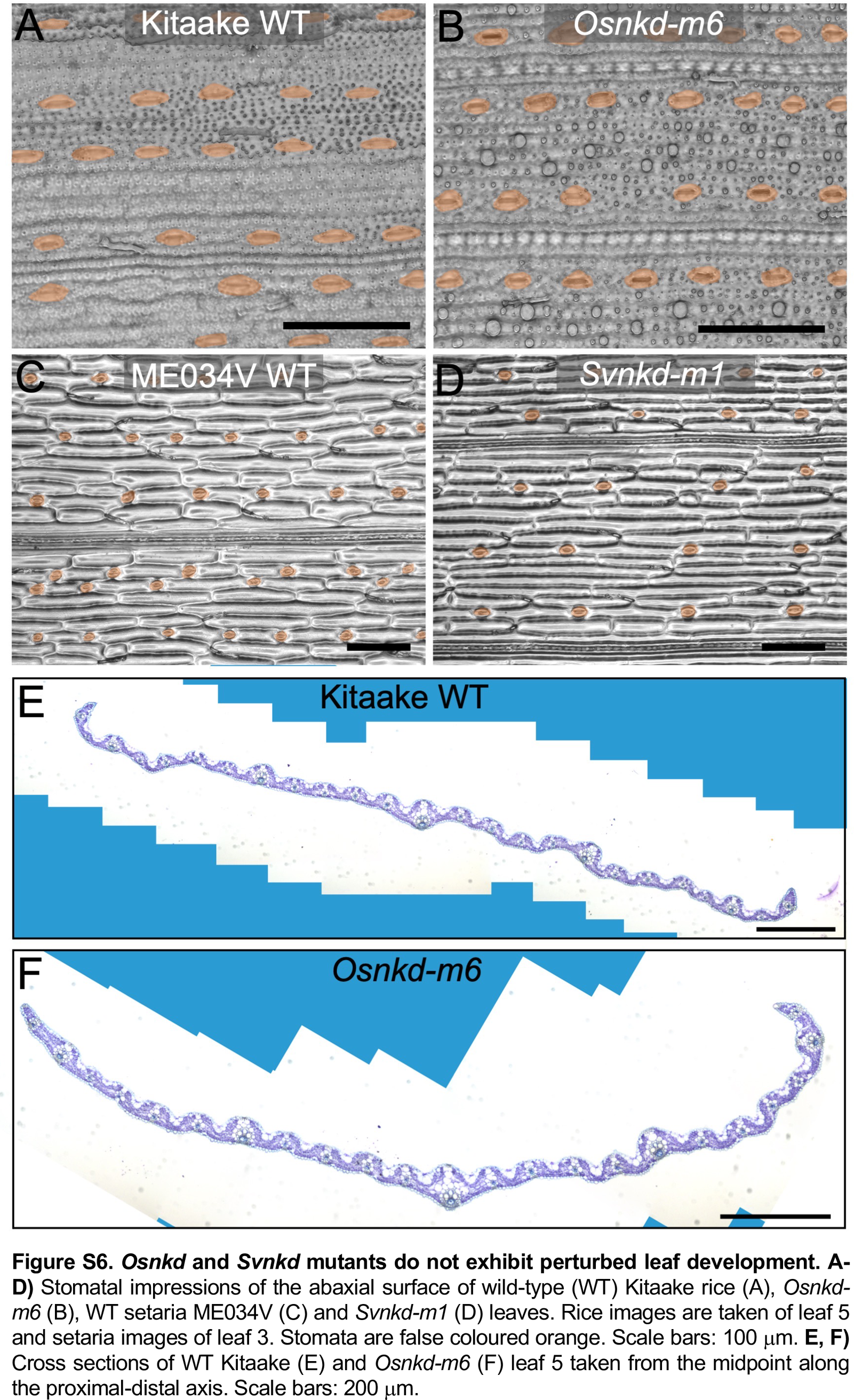

Supplement: S6 Fig — A-D) Stomatal impressions of the abaxial surface of wild-type (WT) Kitaake rice (A), Osnkd-m6 (B), WT setaria ME034V (C) and Svnkd-m1 (D) leaves. Rice images are taken of leaf 5 and setaria images of leaf 3. Stomata are false coloured orange. Scale bars: 100 μm. E, F) Cross sections of WT Kitaake (E) and Osnkd-m6 (F) leaf 5 taken from the midpoint along the proximal-distal axis. Scale bars: 200 μm. (TIF) [file pgen.1010715.s006.tif]

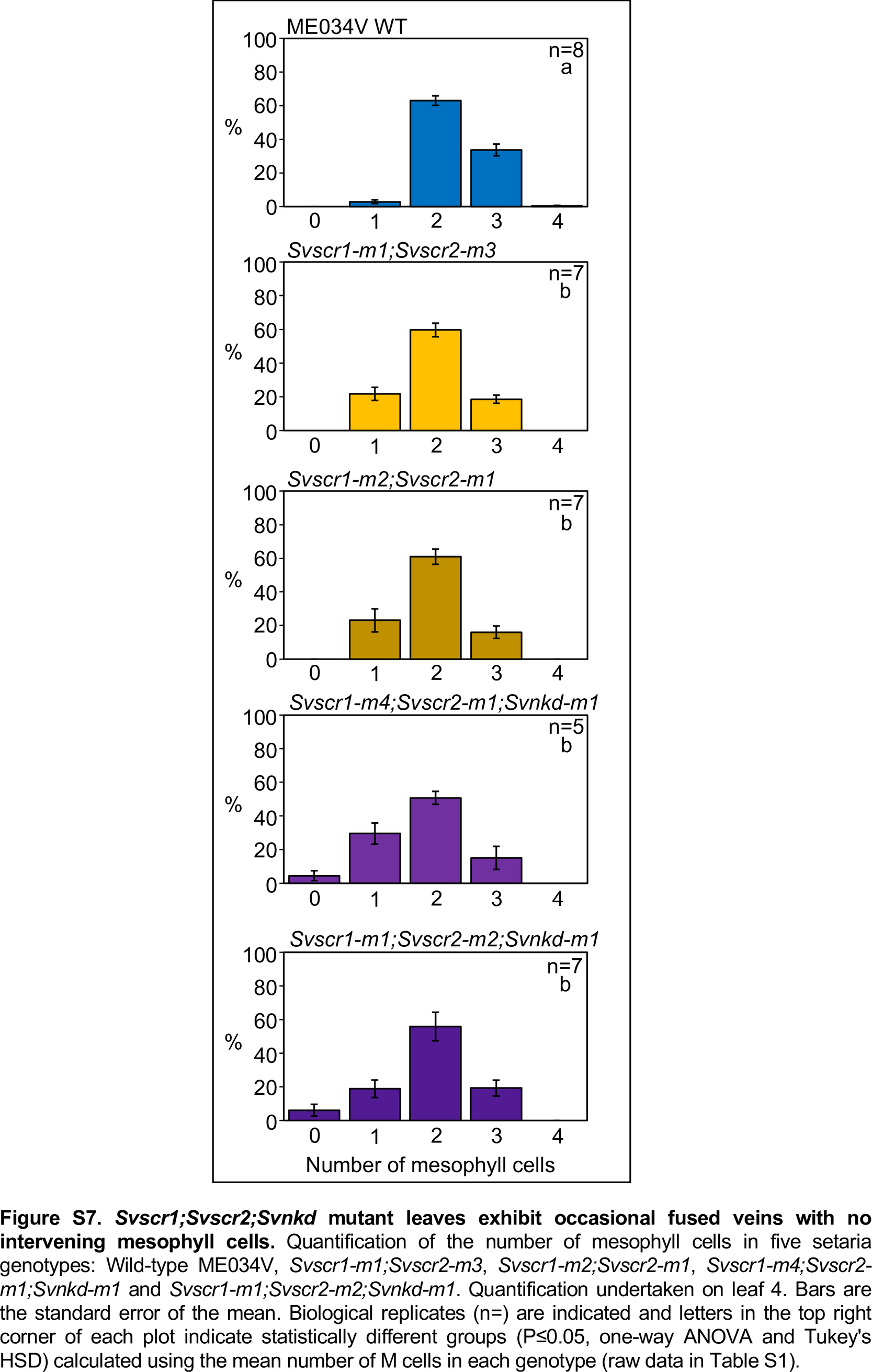

Supplement: S7 Fig — Quantification of the number of M cells in five setaria genotypes: wild-type ME034V, Svscr1-m1;Svscr2-m3, Svscr1-m2;Svscr2-m1, Svscr1-m4;Svscr2-m1;Svnkd-m1 and Svscr1-m1;Svscr2-m2;Svnkd-m1. Quantification undertaken on leaf 4. Bars are the standard error of the mean. Biological replicates (n =) are indicated and letters in the top right corner of each plot indicate statistically different groups (P≤0.05, one-way ANOVA and Tukey’s HSD) calculated using the mean number of M cells in each genotype (raw data in S1 Table). (TIF) [file pgen.1010715.s007.tif]

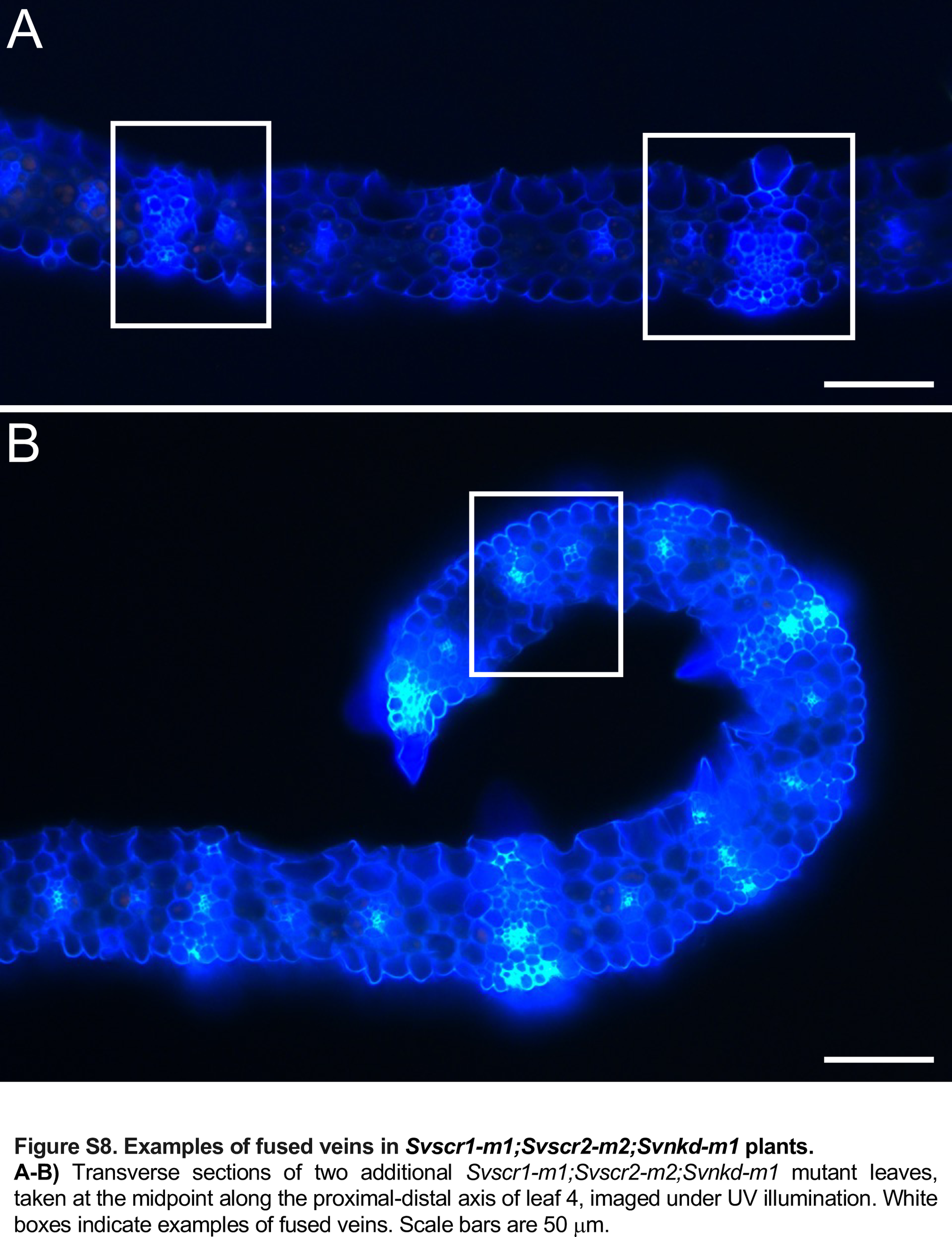

Supplement: S8 Fig — A-B) Transverse sections of two additional Svscr1-m1;Svscr2-m2;Svnkd-m1 mutant leaves, taken at the midpoint along the proximal-distal axis of leaf 4, imaged under UV illumination. White boxes indicate examples of fused veins. Scale bars are 50 μm. (TIF) [file pgen.1010715.s008.tif]

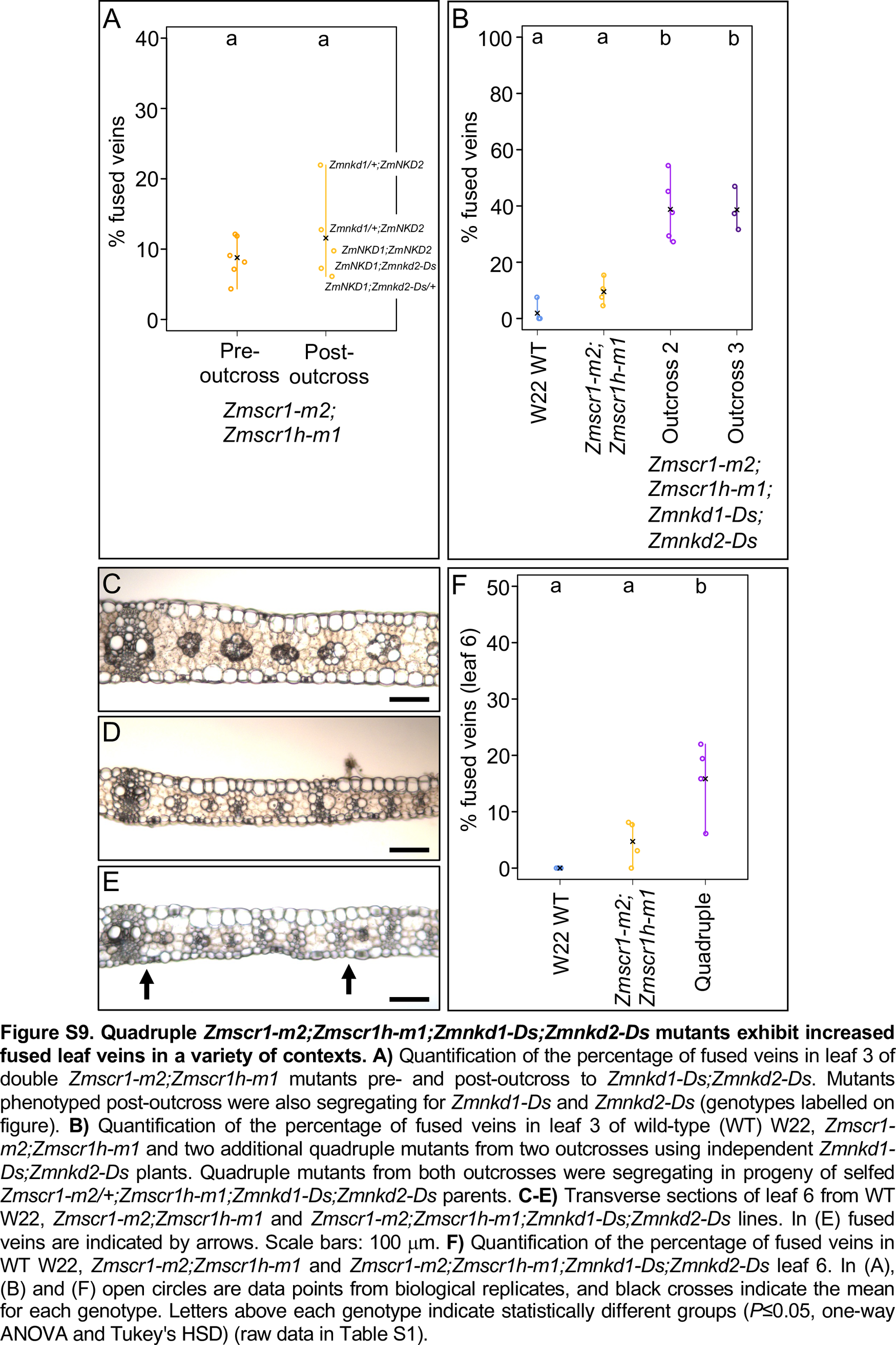

Supplement: S9 Fig — A) Quantification of the percentage of fused veins in leaf 3 of double Zmscr1-m2;Zmscr1h-m1 mutants pre- and post-outcross to Zmnkd1-Ds;Zmnkd2-Ds. Mutants phenotyped post-outcross were also segregating for Zmnkd1-Ds and Zmnkd2-Ds (genotypes labelled on figure). B) Quantification of the percentage of fused veins in leaf 3 of wild-type (WT) W22, Zmscr1-m2;Zmscr1h-m1 and two additional quadruple mutants from two outcrosses using independent Zmnkd1-Ds;Zmnkd2-Ds plants. Quadruple mutants from both outcrosses were segregating in progeny of selfed Zmscr1-m2/+;Zmscr1h-m1;Zmnkd1-Ds;Zmnkd2-Ds parents. C-E) Transverse sections of leaf 6 from WT W22, Zmscr1-m2;Zmscr1h-m1 and Zmscr1-m2;Zmscr1h-m1;Zmnkd1-Ds;Zmnkd2-Ds lines. In (E) fused veins are indicated by arrows. Scale bars: 100 μm. F) Quantification of the percentage of fused veins in WT W22, Zmscr1-m2;Zmscr1h-m1 and Zmscr1-m2;Zmscr1h-m1;Zmnkd1-Ds;Zmnkd2-Ds leaf 6. In (A), (B) and (F) open circles are data points from biological replicates, and black crosses indicate the mean for each genotype. Letters above each genotype indicate statistically different groups (P≤0.05, one-way ANOVA and Tukey’s HSD (raw data in S1 Table). (TIF) [file pgen.1010715.s009.tif]

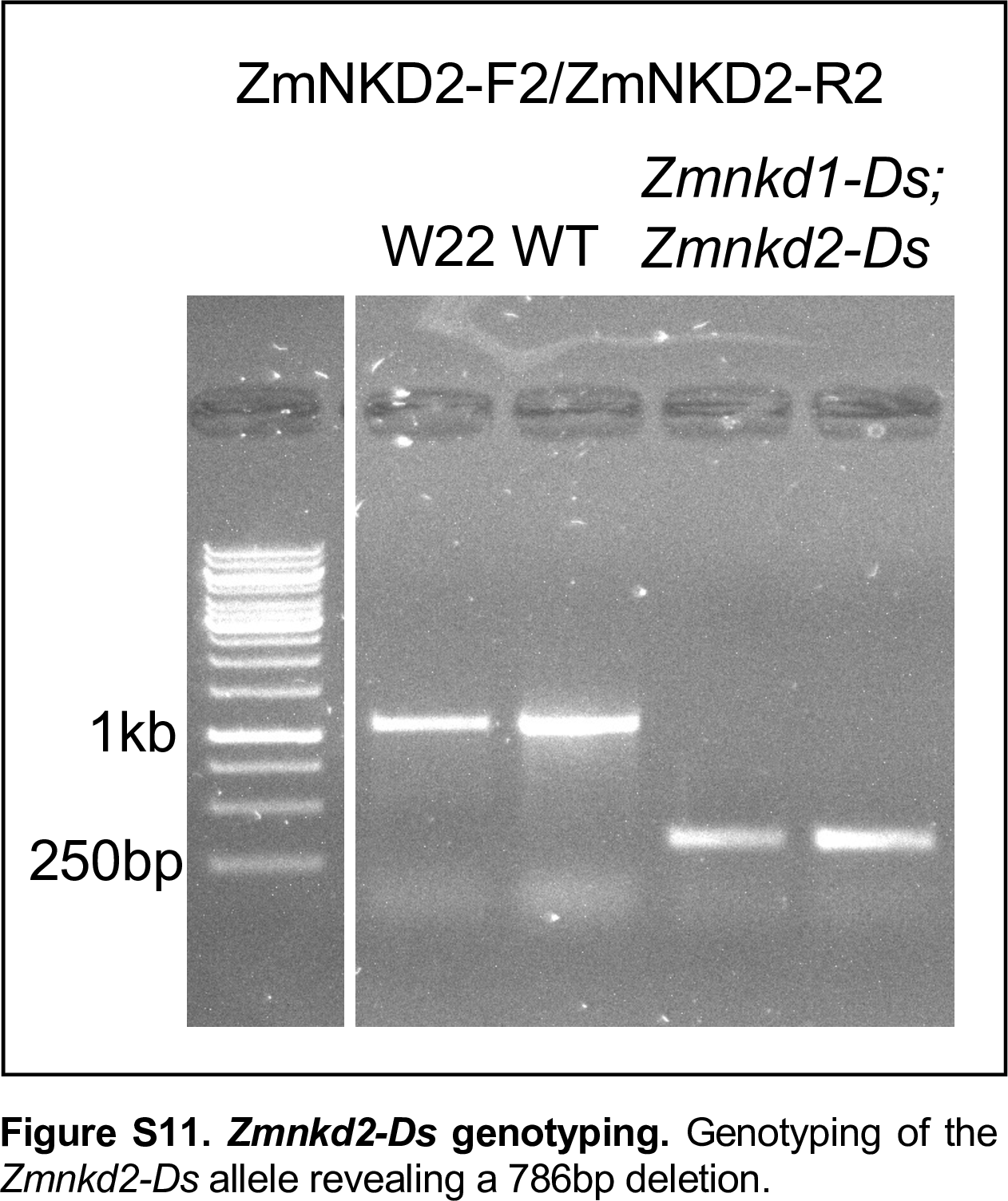

Supplement: S11 Fig — Genotyping of the Zmnkd2-Ds allele revealing a 786bp deletion. (TIF) [file pgen.1010715.s011.tif]
