# Supplementary material for: Mutations in NAKED-ENDOSPERM IDD genes reveal functional interactions with SCARECROW during leaf patterning in C4 grasses
Source: PLoS Genet. 2023 Apr 17;19(4):e1010715. doi: 10.1371/journal.pgen.1010715 (PMC10138192; doi:10.1371/journal.pgen.1010715)
Supplement: S10 Fig — A-C) Cross sections of mature embryos of wild-type W22 (A), double Zmscr1-m2;Zmscr1h-m1 mutants (B) and double Zmnkd1-Ds;Zmnkd2-Ds mutants (C). Double Zmscr1-m2;Zmscr1h-m1 mutants were derived from selfed Zmscr1-m2/+;Zmscr1h-m1 parents whereas double Zmnkd1-Ds;Zmnkd2-Ds mutants were derived from selfed double mutant parents. Three embryos of each genotype are shown. Lateral (L) and developing intermediate (V or V* if very early in development) veins are indicated in the oldest leaf primordium, which in most cases is at plastochron (P) 4. Instances of fused veins are indicated by red lines. D) Cross sections of the double Zmnkd1-Ds;Zmnkd2-Ds mutant embryos in (C) shown at lower magnification to illustrate coleoptile phenotypes. Arrows point to vascular centres in the coleoptile. E, F) Quadruple mutants derived from selfed Zmscr1-m2/+;Zmscr1h-m1;Zmnkd1-Ds/+;Zmnkd2-Ds/+ (nkd heterozygous) (E) or selfed Zmscr1-m2/+;Zmscr1h-m1;Zmnkd1-Ds;Zmnkd2-Ds (nkd homozygous) (F) parents. Labels as for (A-C). (PDF) [file pgen.1010715.s010.pdf]

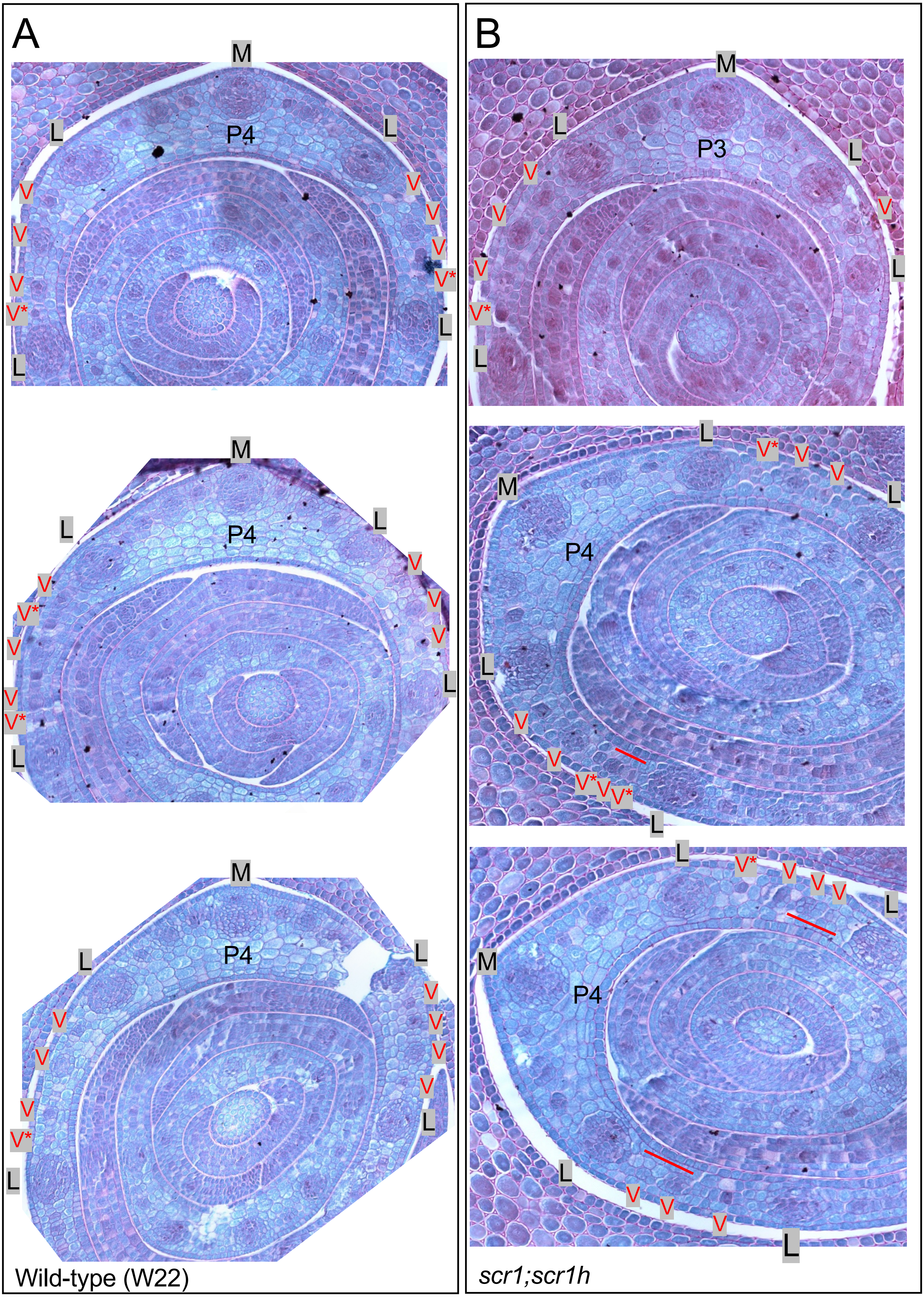

A
M
L
P4
V
V*
Wild-type (W22)
B
M
L
P3
P4
V
V*
scr1;scr1h

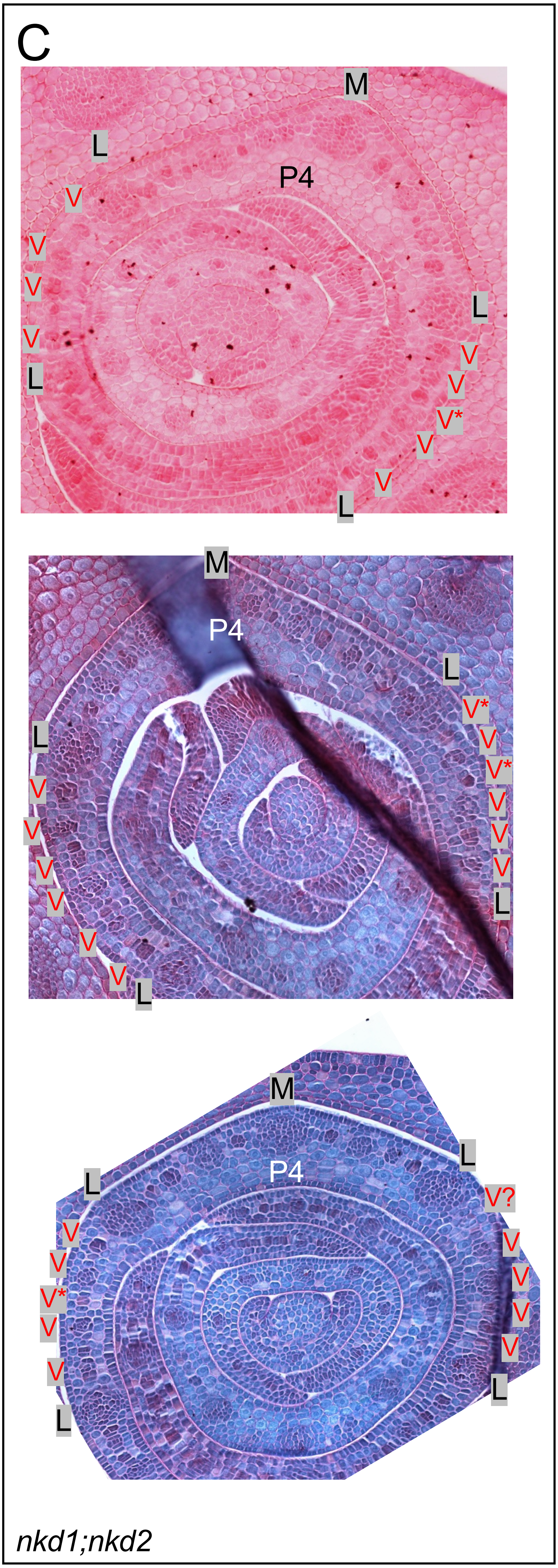
C
M
L
P4
V
V
V
V
L
L
V
V
V*
V
V
L
M
P4
L
V*
V
V*
V
V
V
L
L
V
V
V
V
V
V
L
M
P4
L
L
V?
V
V
V*
V
V
V
V
V
L
L
nkd1;nkd2

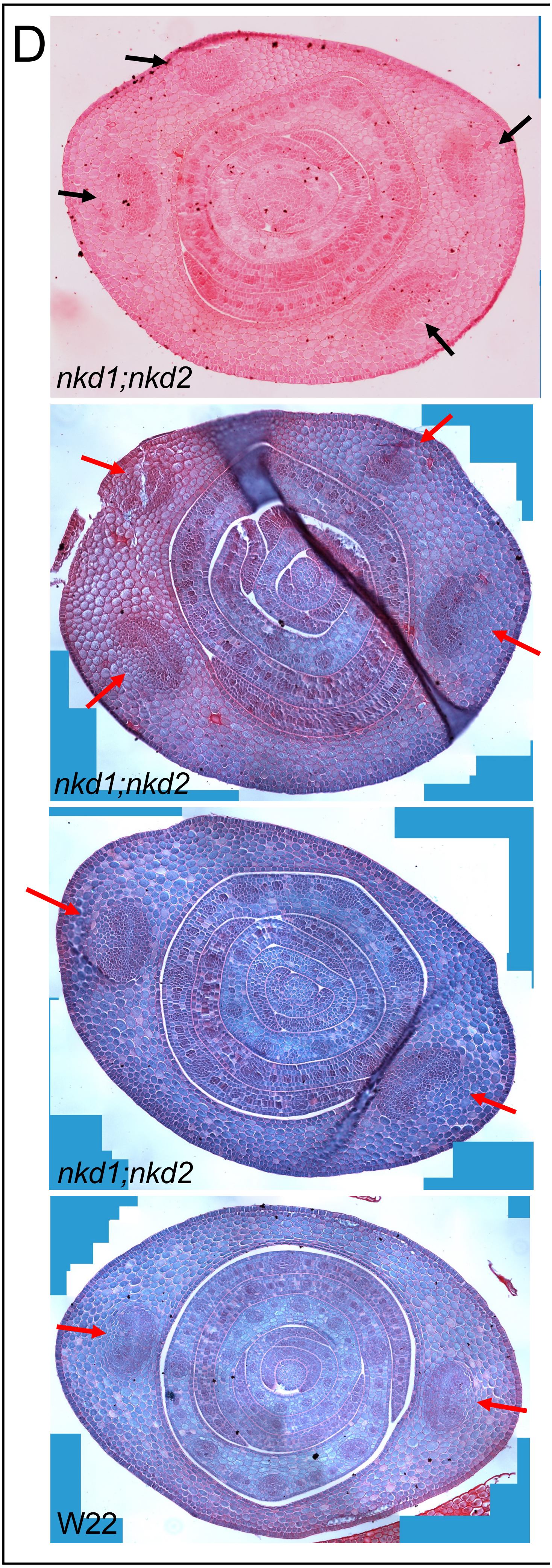
D
nkd1;nkd2
nkd1;nkd2
nkd1;nkd2
W22

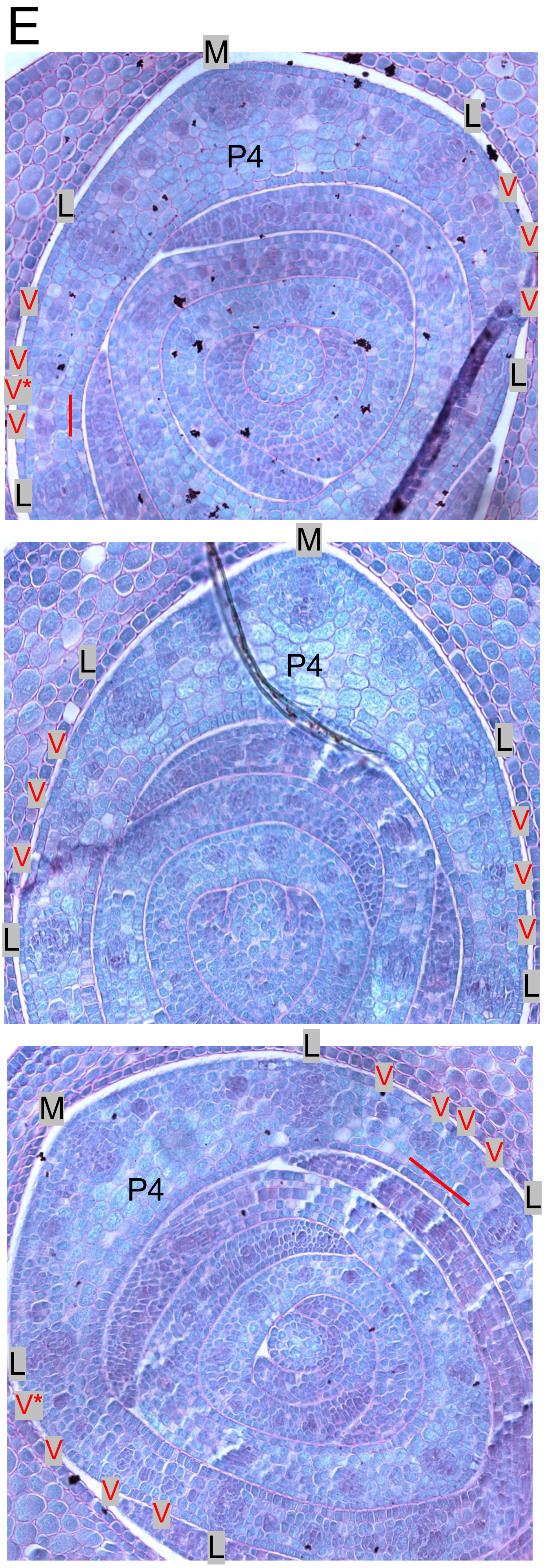


*scr1;scr1h;nkd1;nkd2* (het parent)

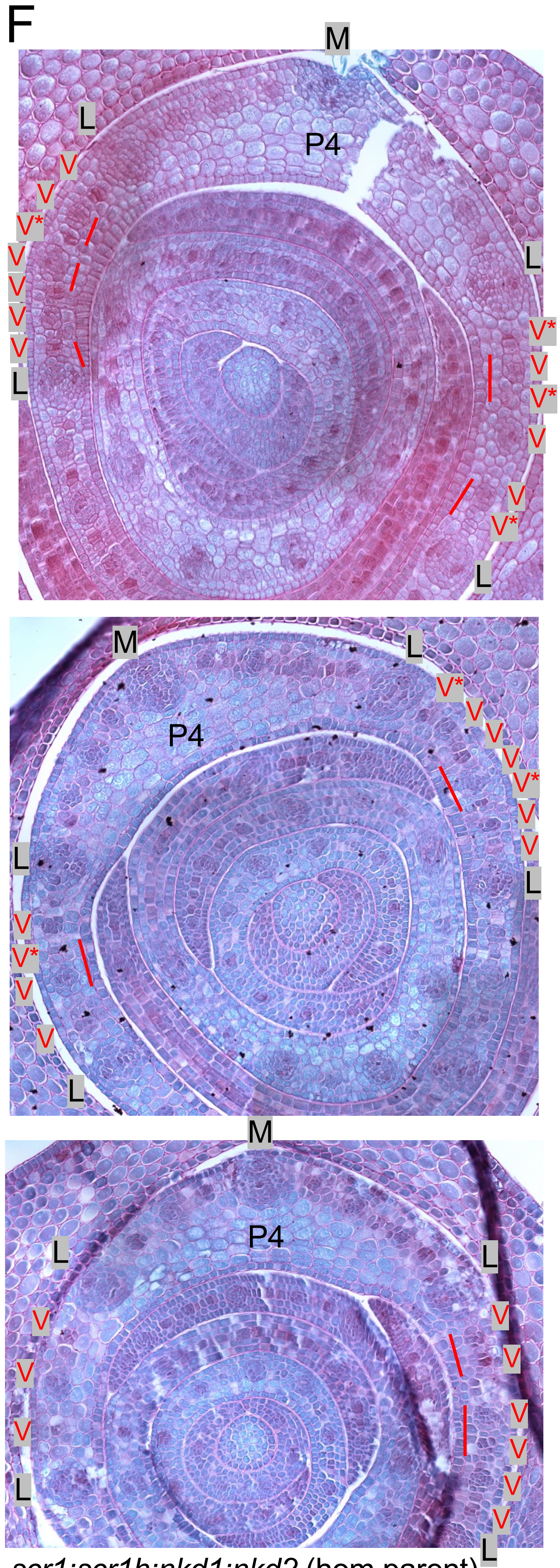


*scr1;scr1h;nkd1;nkd2* (hom parent)

**Figure S10**. Leaf patterning defects are evident in mature embryos of maize. **A-C)** Cross sections of mature embryos of wild-type W22 (A), double *Zmscr1-m2;Zmscr1h-m1* mutants (B) and double *Zmnkd1-Ds;Zmnkd2-Ds* mutants (C). Double *Zmscr1-m2;Zmscr1h-m1* mutants were derived from selfed *Zmscr1-m2/+;Zmscr1h-m1* parents whereas double *Zmnkd1-Ds;Zmnkd2-Ds* mutants were derived from selfed double mutant parents. Three embryos of each genotype are shown. The midvein (M), lateral (L) and developing intermediate (V or V* if very early in development) veins are indicated in the oldest leaf primordium, which in most cases is at plastochron (P) 4. Instances of fused veins are indicated by red lines. **D)** Cross sections of the double *Zmnkd1-Ds;Zmnkd2-Ds* mutant embryos in (C) shown at lower magnification to illustrate coleoptile phenotypes. Arrows point to vascular centres in the coleoptile. **E, F)** Quadruple mutants derived from selfed *Zmscr1-m2/+;Zmscr1h-m1;Zmnkd1-Ds/+;Zmnkd2-Ds/+* (*nkd* heterozygous) (E) or selfed *Zmscr1-m2/+;Zmscr1h-m1;Zmnkd1-Ds;Zmnkd2-Ds* (*nkd* homozygous) (F) parents. Labels as for (A-C).
